# Deep Computerized Adaptive Testing

**DOI:** 10.1017/psy.2026.10106

**Published:** 2026-03-27

**Authors:** Jiguang Li, Robert Gibbons, Veronika Ročková

**Affiliations:** 1 Econometrics and Statistics, The University of Chicago Booth School of Business, USA; 2 Department of Statistics, The University of Chicago, USA

**Keywords:** adaptive data collection, Bayesian methods, deep Q-learning, multidimensional item response theory

## Abstract

Computerized adaptive tests (CATs) play a crucial role in educational assessment and diagnostic screening in behavioral health. Unlike traditional linear tests that administer a fixed set of pre-assembled items, CATs adaptively tailor the test to an examinee’s latent trait level based on their previous responses. We introduce a novel CAT system that builds on recent advances in Bayesian multivariate IRT. Our approach leverages direct sampling from the latent factor posterior distributions, significantly accelerating existing information-theoretic item-selection methods by eliminating the need for computationally intensive Markov chain Monte Carlo simulations. To address the potential suboptimality of one-step-ahead item-selection rules, we also develop a double deep Q-learning algorithm that efficiently learns an optimal item-selection policy offline using a calibrated item bank. Through simulation and real-data studies, we demonstrate that our approach not only accelerates existing item-selection methods but also highlights the potential of reinforcement learning (RL) in CATs. Notably, our Q-learning-based strategy consistently achieves the fastest posterior variance reduction, leading to earlier test termination. These results demonstrate the promise of combining exact posterior sampling with RL to deliver scalable, high-precision CATs.

## 1 Introduction

Multidimensional computerized adaptive testing (MCAT) has revolutionized the field of educational and psychological assessments by dynamically selecting tailored items from a large test pool, thereby enhancing the efficiency and precision of latent ability estimates (van der Linden & Glas, 2010). Powered by multidimensional item response theory (MIRT; Bock & Gibbons, 2021), MCAT leverages multidimensional statistical inference to evaluate respondents’ multidimensional latent traits and allows for more comprehensive and efficient assessments compared to unidimensional approaches. MCAT’s adaptability and accuracy are also particularly crucial in high-stakes diagnostic assessments, such as in clinical psychology and psychiatry, where it can be substituted for in-person assessments by clinical professionals, especially in areas with limited medical resources (Gibbons et al., 2016).

A substantial body of research in MCAT has focused on item-selection strategies derived from experimental design principles. One prominent strategy is to select items that maximize the determinant of the Fisher information matrix evaluated at the current estimates of the latent traits (Segall, 1996, 2000), known as the D-optimality criterion. An alternative, the A-optimality criterion, aims to minimize the trace of the asymptotic covariance matrix, thereby reducing overall estimation variance (van der Linden, 1999). In the absence of nuisance latent abilities, both A-optimality and D-optimality have demonstrated superior accuracy relative to other common experimental design criteria (Mulder & van der Linden, 2009).

Another prominent line of MCAT item-selection algorithms leverages Kullback–Leibler (KL) information, often within a Bayesian framework. A common approach is to select items that produce response distributions at the true latent trait value, 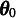 , that differs maximally from the response distributions generated at the other value of 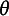 (Chang & Ying, 1996; Veldkamp & van der Linden, 2002). Moving beyond response distributions alone, some researchers propose maximizing the KL divergence between the current posterior distribution and the posterior distribution at the next selection step, thereby enhancing adaptation through updated trait estimates (Mulder & van der Linden, 2010). Another promising strategy is the mutual information (MI) criterion, which aims to maximize entropy reduction of the current posterior distribution, encouraging more and more accurate posterior estimates (Weissman, 2007). In particular, Wang and Chang (2011) demonstrate both theoretical and empirical advantages of the Bayesian MI item-selection rule over common experimental criteria such as D-optimality. More detailed theoretical comparison of KL information and Fisher information criteria is presented by Wang et al. (2011).

Although numerous effective item-selection rules have been proposed in the MCAT literature, they all rely on one-step lookahead optimization of an information-theoretic criterion. Despite their ease of implementation and attractive theoretical properties, these selection rules are inherently myopic: they select items based solely on immediate information gain, ignoring how current choices influence future decisions, which can lead to suboptimal policies. For example, existing methods tend to favor items with high loading parameters (Chang, 2015). However, CAT researchers also recommend reserving such items for the later stages of testing to improve efficiency (Chang & Ying, 1999). Integrating heuristic guidance into existing selection rules remains challenging.

Addressing these limitations, we propose a novel deep CAT system that integrates a flexible Bayesian MIRT model with a non-myopic online item-selection policy, guided by reinforcement learning (RL) principles (Sutton & Barto, 2018). Leveraging recent advancements in Bayesian sparse MIRT (Li et al., 2025), our framework seamlessly accommodates multiple latent factors with complex loading structures, while maintaining scalability in both the number of items and factors. To learn the optimal item-selection policy that prioritizes the assessment of target factors, we draw on contemporary RL methodologies and introduce a general double deep Q-learning algorithm (Mnih et al., 2015; van Hasselt et al., 2016). This algorithm efficiently trains a Q-network offline using only item parameter estimates; the learned network can then be deployed online to select optimal items based on the current multivariate latent factor posterior distribution. When the test terminates, our framework robustly characterizes the full latent factor posterior distributions rather than providing only a point estimate.

A primary contribution of our work is to show how the identification of the latent factor posterior distribution leads to substantial computational gains in online item selection. Given that such posterior distribution is deemed to be non-Gaussian and unknown, traditional Bayesian methods typically rely on expensive Markov chain Monte Carlo (MCMC) simulations (Béguin & Glas, 2001) and combined with additional data augmentation to handle categorical likelihood (Albert & Chib, 1993; Polson et al., 2013). Our approach achieves substantial acceleration by directly sampling latent factor posterior distributions (Botev, 2016; Durante, 2019; Li et al., 2025). Notably, this improvement not only increases the efficiency of existing Bayesian item-selection procedures but also provides a computational foundation for training our proposed Q-learning algorithm through rapid, large-scale simulations of testing sessions.

Another critical advancement in our work is the integration of CAT within an RL framework. This approach addresses the practical need to prioritize accurate estimation and to overcome known limitations of greedy item-selection methods in sequential decision-making (Bertsekas & Tsitsiklis, 1996). Building on the Bayesian MIRT foundation, our neural-network architecture incorporates the identified posterior parameters as state variables, allowing the model to learn optimal item-selection policies through a large amount of testing simulations. This formulation bridges the two paradigms: the Bayesian component provides statistically grounded representations of examinee uncertainty, while the RL component leverages these representations to optimize sequential decisions. The trained neural network is deployable on standard laptops without GPU acceleration, making it suitable for online adaptive testing applications. The sequential nature of CAT aligns naturally with deep Q-learning methods, which have demonstrated remarkable success across diverse application domains (Kalashnikov et al., 2018; Silver et al., 2016). Notably, RL has been successfully employed in educational measurement settings to design personalized learning plans (Li et al., 2023; Tan et al., 2020).

Finally, our work aligns with the emerging research trend of framing traditional statistical sequential decision-making problems as optimal policy learning tasks (Rainforth et al., 2024). This perspective has appeared in Bayesian adaptive design (Chaloner & Verdinelli, 1995; Foster et al., 2021; Sebastiani & Wynn, 2000) and Bayesian optimization (Lam et al., 2016; Srinivas et al., 2010), where recent methods aim to move beyond one-step criteria toward policy learning that account for long-term consequences. Our contribution fits within this broader trend by providing a principled approach to cognitive and behavioral assessment.

The remainder of the article is organized as follows. Section 2 motivates our deep CAT framework using a cognitively complex item bank from a recent clinical study (Gibbons et al., 2024). Section 3 reviews existing information-theoretic item-selection methods and outlines the necessity to reformulate CAT as an RL task. Section 4 introduces a general Bayesian framework that accelerates existing CAT item-selection rules and serves as the foundation for our Q-learning algorithm. Section 5 details our RL approach, including the neural network architecture and double Q-learning algorithm. Finally, Sections 6 and 7 evaluate our method through both simulations and real data experiments.

## 2 Adaptive high-dimensional cognitive assessment

Our proposed deep CAT system is motivated by the growing need for adaptive cognitive assessment in high-dimensional latent spaces. Cognitive impairment, particularly Alzheimer’s dementia (AD), is a major public health challenge, affecting 6.9 million individuals in the United States in 2024 (Alzheimer’s Association, 2024). Early detection of mild cognitive impairment (MCI), a precursor to AD, is crucial for slowing disease progression and improving patient outcomes (Huang et al., 2018). However, traditional neuropsychological assessments are costly, time-consuming, and impractical for frequent use, underscoring the need for more efficient and scalable assessment methods.

Recently, pCAT-COG, the first computerized adaptive test (CAT) item bank based on MIRT for cognitive assessment, demonstrated its potential as an alternative to clinician-administered evaluations (Gibbons et al., 2024). The data were collected from 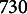 participants. After careful item calibration and model selection, the final item bank consisted of 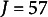 items covering five cognitive subdomains: episodic memory, working memory, executive function, semantic memory, and processing speed. Since each item comprised three related tasks, we used a binary score, where 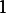 indicates correct answers on all tasks for our analysis.

**Figure 1:**
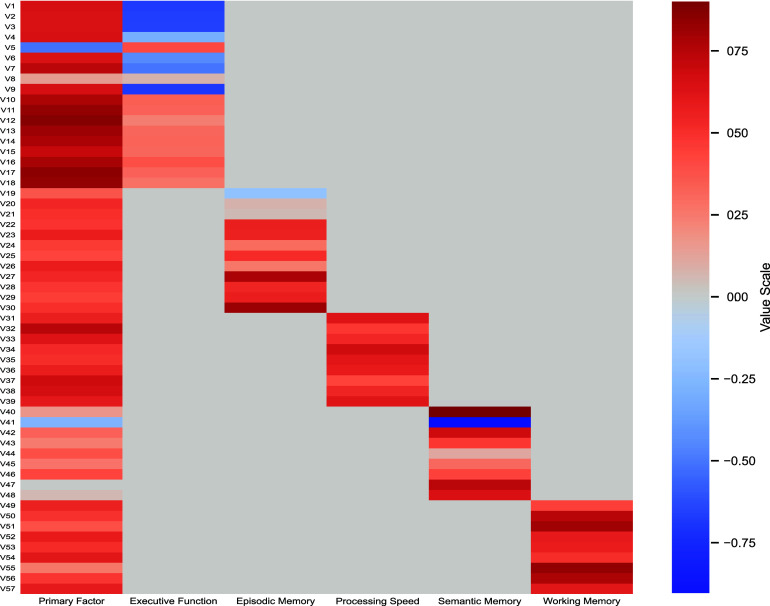
Estimated bifactor factor loading matrix for pCAT-COG.

Following Gibbons et al. (2024), we fit a six-factor bifactor model (Gibbons & Hedeker, 1992) to the 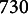 by 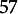 binary response matrix, with one primary factor representing the global cognitive ability and five secondary factors. This yields a 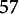 by 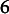 factor loading matrix, visualized in Figure 1, where rows correspond to pCAT-COG items and columns represent distinct factors.

Since items in pCAT-COG are primarily designed to measure the general cognitive factor and only partially capture subdomain information, our goal is to develop an item-selection strategy that efficiently estimates the primary factor (first column) using as few items as possible while maintaining robustness to subdomain influences. Additionally, the selection algorithm must be computationally efficient to navigate a six-dimensional latent space in real time for seamless interactive testing. Online item selection is critical, as learning an optimal sequence from 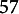 items offline presents an intractable combinatorial problem, even in a binary response setting.

Adaptive cognitive assessment is essential for cognitive assessment, as designing items is costly and administering all items is time-consuming. Our deep CAT system requires on average 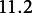 items to reduce the posterior variance of the primary factor from 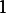 to 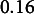 (posterior s.d. 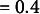 ), whereas the next-best MI method requires 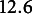 items to achieve the same precision. The item bank is currently expanding to 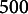 items with a more nationally representative sample. Success on this prototype dataset paves the way for broader deployment in clinical research.

## 3 From one-step optimization to policy learning

We formally define the problem of designing a CAT system from an RL perspective. Typical CAT systems consist of two components:

- *Offline calibration*: An MIRT model is fitted to a calibrated dataset 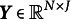 to estimate the item characteristic parameters, where *N* represents the number of examinees, and *J* represents the number of items in the item bank.
- *Online deployment*: Given the estimated item parameters, an item-selection algorithm is deployed online to adaptively select items for future examinees.

The performance of CAT can be measured by the number of items required to estimate an examinee’s latent traits with sufficient precision.

### 3.1 Notation and problem formulation

For the entirety of the article, we assume that the calibration dataset 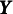 is binary, where each element 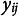 represents whether subject *i* answered item *j* correctly. We consider a general two-parameter MIRT framework with *K* latent factors (Bock & Gibbons, 2021). Let 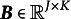 denote the factor loading matrix, and 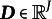 denote the intercept vector. Throughout the article, boldface notation is used to denote vectors and matrices (e.g., 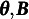 , and 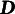 ), while scalar quantity, such as 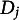 , is written in standard font. For each examinee *i* with multivariate latent trait 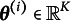 , the data-generating process for 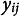 is given by 
(3.1)

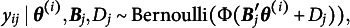

where 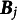 is the *j*th row of 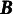 , 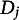 is the *j*th entry of 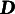 , and 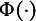 denotes the standard normal cumulative distribution function. The item parameters can be compactly expressed as 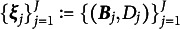 . This two-parameter MIRT framework is highly general, imposing no structural constraints on the loading matrix and requiring no specific estimation algorithms for item parameter calibration.

Given the estimated item parameters, we need to design an item-selection algorithm that efficiently tests a future examinee with unobserved latent ability 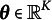 . The sequential nature of the CAT problem makes Bayesian approaches particularly appealing. Without loss of generality, we assume a standard multivariate Gaussian prior on 
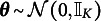
 and introduce the following notation:

- For any positive integer *n*, let 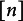 denote all the positive integers no greater than *n*. Let 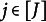 be the index for the items in the item bank.
- Let 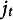 denote the index of the item selected at step *t* based on an arbitrary item-selection rule. Define 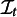 as the set of the first *t* administered items, and let 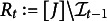 , the set of available items before the *t*th item is picked.
- We use the shorthand notation 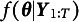 to represent the latent factor posterior distributions 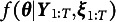 after *T* items have been selected. Here, the response history is denoted by 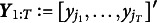 , and the item parameters are given by 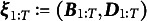 , where 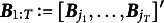 and 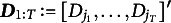 .

At time 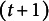 , the algorithm takes the current posterior 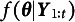 as input and outputs the next item selection 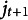 . This process continues until at time 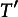 , either when the posterior variance of 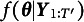 falls below a predefined threshold 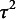 , or when 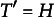 , where 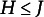 is the maximum number of items that can be administered. Hence, the goal of CAT is to minimize 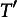 given the estimated item parameters of the item bank.

### 3.2 Reviews of KL information item-selection rules

Given that our proposed deep CAT system is built on a general Bayesian MIRT framework (Li et al., 2025), we briefly revisit common Bayesian item-selection rules and show in Section 4.1 how our framework can be used to accelerate these baseline methods. The popular KL expected a priori (EAP) rule selects the *t*th item based on the average KL information between response distributions on the candidate item at the EAP estimate 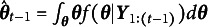 , and random factor 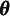 sampled from the posterior distribution 
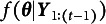
 (Veldkamp & van der Linden, 2002): 
(3.2)

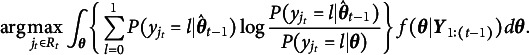



Rather than focusing on the KL information based on the response distributions, Mulder and van der Linden (2010) propose the MAX Pos approach by maximizing the KL information between two subsequent latent factor posteriors 
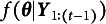
 and 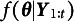 . Intuitively, this approach prioritizes items that induce the largest shift in the posterior, formalized as 
(3.3)

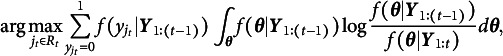

where 
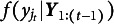
 represents the posterior predictive probability: 
(3.4)

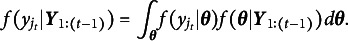

A third Bayesian strategy, the MI approach, maximizes the MI between the current posterior distribution and the response distribution of new item 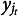 (Weissman, 2007). Rooted in experimental design theory (Rényi, 1961), we can also interpret MI as the entropy reduction of the current posterior distribution of 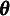 after observing new response 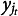 . More formally, 
(3.5)

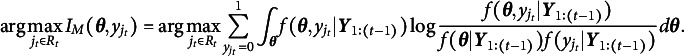



Given the impressive empirical success of the MI method (Wang & Chang, 2011), we also introduce a competitive heuristic item-selection rule that chooses items with the highest predictive variance under the current posterior estimates, serving as an additional benchmark method. Formally, write 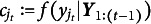 as defined in (3.4), and consider the following criterion: 
(3.6)

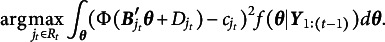

This rule selects the item 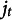 that maximizes the variance of the predictive means, weighted by the current posterior 
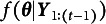
 . In Appendix A of the Supplementary Material, we establish a connection between this selection rule and the MI method, showing that both favor items with high prediction uncertainty.

### 3.3 Reinforcement learning formulation

Existing item-selection rules face several limitations: first, these methods rely on one-step-lookahead selection, choosing items that provide the most immediate information without considering their impact on future selections, which can result in suboptimal policies (Sutton & Barto, 2018). Second, they are heuristically designed to balance information across all latent factors, rather than emphasizing the most essential factors of interest. Finally, these rules do not directly minimize test length, potentially increasing test duration without proportional gains in accuracy.

A more principled approach is to formulate item selection as an RL problem, where the optimal policy is learned using Bellman optimality principles rather than relying on heuristics that ignore long-term planning. Beyond addressing myopia, an RL-based formulation provides a direct mechanism to minimize the number of items required to reach a predefined posterior variance reduction threshold, explicitly guiding item selection toward accurately measuring the primary factors of interests.

More generally, consider a general finite horizon setting where each examinee can answer at most 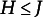 items, and the CAT algorithm terminates whenever the posterior variance of all factors of interest is smaller than a certain threshold 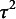 , or when *H* is reached. Since *H* can be set sufficiently large, it serves as a practical secondary stopping criterion to prevent excessively long tests. Formally:

- **State space** 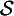 : The space of all possible latent factor posteriors 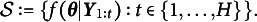 The state variable in CAT represents the Bayesian estimate of the examinee’s latent traits as a multivariate distribution at time *t*. We may write 
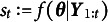
 , with 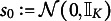 . The state space is discrete and can be exponentially large. Since there are *J* items in the item bank and the responses are binary, the total number of possible states is 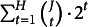 .
- **Action space** 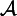 : The item bank 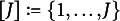 . However, at the *t*th selection time, the action space is the remaining items in the test bank that have not yet been selected 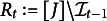 , as we do not select the same item twice.
- **Transition kernel** 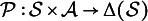 , where 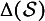 denotes the space of probability distributions over 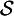 . The transition kernel 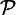 specifies the stochastic rule that governs how the state evolves after an action is taken. In the CAT context, the kernel maps the current latent-trait estimate 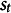 and the selected item 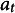 to the next posterior state 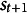 . Conditional on 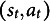 , the next estimate 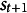 depends on the examinee’s binary response 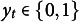 to item 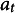 , which yields only two possible posterior updates. Because the true ability of the examinee and thus the response probability 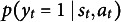 are unknown, the transition kernel in CAT is also unknown and must be approximated.
- **Reward function**: To directly minimize the test length, we assign a simple 0–1 reward structure, where we assign negative rewards whenever more items are needed to reduce posterior variance to a given threshold. In a *K*-factor MIRT model, we often prioritize a subset of factors 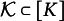 (e.g., 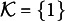 for pCAT-COG), with the test terminating once the posterior variances of all factors in 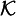 fall below the predefined threshold 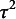 : 
(3.7)

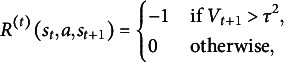

where 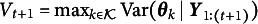 is the maximum marginal posterior variance among the prioritized factors 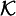 . This also simplifies the learning of the value function, as the rewards are always bounded integers within 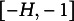 . We further illustrate the advantages of adopting this reward structure in Appendix E of the Supplementary Material.

Rather than following a heuristic rule, we learn a policy 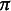 that maps the current state (latent factor estimates) to a distribution over potential items: 

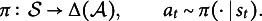

Given an initial state 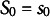 and the discount factor 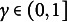 , the value function is 
(3.8)

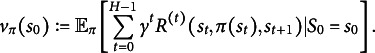

The expectation 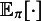 is taken over trajectories generated by drawing 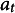 from 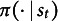 and then 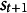 from the transition kernel 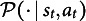 . Because direct evaluation of (3.8) is challenging, it is more convenient to consider the action-value function 
(3.9)

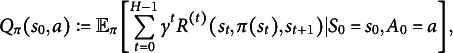

with 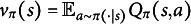 . The optimal policy 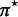 satisfies the Bellman equation (Bertsekas & Tsitsiklis, 1996): 
(3.10)

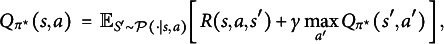

where 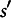 is the next posterior estimate after applying action *a* to the current posterior *s*, and 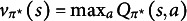 . Since the state space grows exponentially and the transition kernel is unknown, solving for 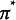 using traditional dynamic programming approaches becomes intractable (Sutton & Barto, 2018).

In practice, our deep Q-learning approach (Mnih et al., 2013) does not evaluate these expectations analytically. Let the transition 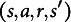 represent one testing step, where item *a* is selected under state *s*, the reward *r* is observed, and the posterior is updated to 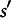 through the Bayesian MIRT model. We approximate the Bellman fixed point in (3.10) by fitting a parametric function 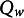 that minimizes the squared temporal-difference loss: 
(3.11)

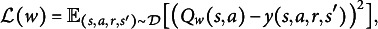

where 
(3.12)

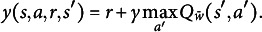

Here, 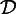 (replay buffer) denotes a collection of previously observed testing steps 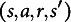 generated during simulation, which is used to approximate the expectation above by empirical averaging. The parameter 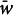 corresponds to a delayed copy of the model parameters *w*, updated less frequently to stabilize the numerical optimization. We describe the full Q-learning algorithm in Section 5.

## 4 Accelerating item selection via posterior identification

The central insight of our deep CAT framework is that, by iteratively applying the E-step of the PXL-EM algorithm (Li et al., 2025), the latent factor posteriors admit tractable posterior updates. This result is critical for RL, as the posterior distribution is deemed to be non-Gaussian and is analytically intractable under the traditional MIRT literature. By obtaining a tractable representation of this posterior, we can parameterize the examinee’s evolving ability and uncertainty as a well-defined state variable, which is an essential prerequisite for applying Q-learning to adaptive testing. The Bayesian MIRT formulation thus not only replaces costly MCMC procedures with efficient posterior updates under a probit link for existing Bayesian item-selection rules discussed in Section 3.2, but also supplies the statistical foundation that makes the subsequent RL framework feasible.

Specifically, we show that the latent factor posterior updates during CAT belong to an instance of the unified skew-normal distribution (Arellano-Valle & Azzalini, 2006), defined as follows.

Definition 4.1. Let 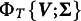 represent the cumulative distribution function of a *T*-dimensional multivariate Gaussian distribution 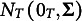 evaluated at vector 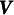 . A *K*-dimensional random vector 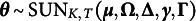 has the *unified skew-normal* distribution if it has the probability density function: 

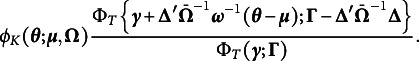

Here, 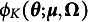 is the density of a *K*-dimensional multivariate Gaussian with expectation 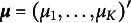 , and a *K* by *K* covariance matrix 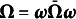 , where 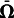 is the correlation matrix and 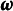 is a diagonal matrix with the square roots of the diagonal elements of 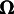 in its diagonal. 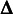 is a *K* by *T* matrix that determines the skewness of the distribution, and 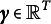 controls the flexibility in departures from normality.

In addition, the 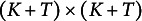 matrix 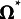 , having blocks 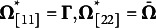 , and 
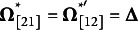
 , needs to be a full-rank correlation matrix.

Suppose an arbitrary CAT item-selection algorithm has already selected *T* items with item parameters 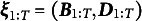 , where 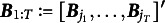 and 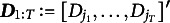 . Then it is possible to show the following result.

Theorem 4.1. Consider a K-factor CAT item-selection procedure after selecting *T* items, with 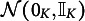 prior placed on the test taker’s latent trait 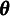 . If 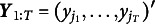 is conditionally independent binary response data from the two-parameter probit MIRT model defined in (3.1), then 

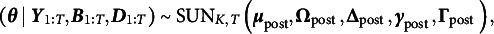

with posterior parameters 

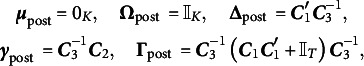

where 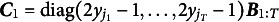 and 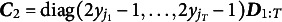 . The matrix 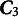 is a *T* by *T* diagonal matrix, where the 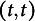 th entry is 
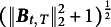
 , with 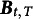 representing the *t*th row of 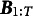 .

Theorem 4.1 provides an exact finite-sample Bayesian characterization of the latent factor posterior. With a multivariate normal prior on 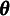 and a probit MIRT likelihood, the posterior 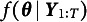 belongs to the unified skew-normal (SUN) family. This does not conflict with the empirical Bayes result of Chang and Stout (1993), which establishes asymptotic posterior normality as the number of items *J* grows; related discussion of Bayesian latent-trait estimation and asymptotic covariance properties in MIRT is given in Wang (2015). The finite-sample form is particularly relevant for CAT, where only a small number of items have been administered and large-sample normal approximations may be unreliable. This representation enables exact posterior calculations, improving uncertainty quantification and item selection in short tests. For ordinal responses under a probit link, closely related SUN-based approximations can also be developed as discussed in Li et al. (2025). The latent factor posterior can often be well approximated within the SUN family, although the representation is generally not exact.

According to Arellano-Valle and Azzalini (2006), an arbitrary unified skew-normal distribution 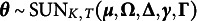 has a stochastic representation as a linear combination of a *K*-dimensional multivariate normal random variable 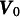 , and a *T*-dimensional truncated multivariate normal random variable 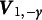 as follows: 
(4.1)

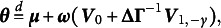

where 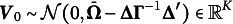 , and 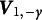 is a multivariate normal distribution 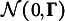 truncated to 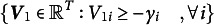 .

Based on Theorem 4.1, we have a closed-form expression for the posterior parameters 
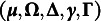
 for 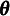 . Recall that 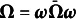 is the standard covariance correlation matrix decomposition from Definition 4.1, and hence the only unrealized stochastic terms in Equation (4.1) are 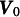 and 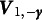 . This suggests that sampling from the latent factor posterior distribution 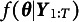 requires two independent steps: sampling from a *K*-dimensional multivariate normal distribution 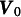 and a *T*-dimensional multivariate truncated normal distribution 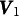 . As a result, the direct sampling approach scales efficiently with the number of factors *K*, since generating samples from *K*-dimensional multivariate normal distributions is trivial. Moreover, in CAT settings, the test is typically terminated early, meaning *T* remains relatively small. When *T* is moderate (e.g., 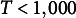 ), sampling from the truncated multivariate normal distribution remains computationally efficient using the minimax tilting method (Botev, 2016). The exact proof of Theorem 4.1 and the sampling details can be found in Appendix B of the Supplementary Material.

The direct sampling approach provides substantial gains in both computational efficiency and numerical precision compared with traditional MCMC methods commonly used in the MIRT literature (Béguin & Glas, 2001; Jiang & Templin, 2019). In standard MIRT settings, the posterior distribution of the latent factors is typically regarded as intractable and non-Gaussian, requiring a Markov chain to be constructed via data-augmentation techniques (Albert & Chib, 1993; Polson et al., 2013) so that its stationary distribution approximates the posterior. This procedure entails repeated simulation of augmented data and is inherently sequential, which limits opportunities for parallelization. Moreover, it demands additional tuning, burn-in, and convergence diagnostics to ensure that the chain adequately converges to the posterior distribution. In contrast, Theorem 4.1 establishes that the posterior distribution can be expressed exactly as a unified skew-normal distribution, allowing direct and parallel sampling without the need for iterative convergence procedures.

Theorem 4.1 also plays a central role in our proposed deep Q-learning algorithm, as it fully characterizes the latent factor posterior distribution 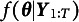 , enabling the parameterization of the state variable 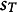 , which serves as an input to the Q-network illustrated in Section 5.1. We illustrate how Theorem 4.1 can be used to accelerate existing item-selection rules, and the same idea can be extended to fully Bayesian item-selection settings as considered by van der Linden and Ren (2020).

### 4.1 Accelerating existing rules

While Bayesian CAT criteria require multidimensional integration, these quantities are typically evaluated in practice via Monte Carlo approximation rather than analytic quadrature in moderate to high dimensions. In such Bayesian CAT implementations, the dominant computational cost lies in obtaining valid samples from the latent factor posterior. By providing exact posterior samples without Markov chain construction, our approach removes this primary bottleneck and renders the remaining Monte Carlo integration computationally efficient and easily parallelizable. For example, in computing the KL-EAP item-selection rule (3.2), we directly sample from 
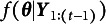
 instead of resorting to MCMC and evaluate the integral via Monte Carlo integration. Since the EAP estimate 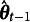 remains fixed at time step *t*, evaluating the KL information term is straightforward. For the Max Pos item-selection rule in Equation (3.3), we again obtain i.i.d. samples from 
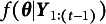
 and compute the posterior predictive probabilities in (3.4). Although the density ratio 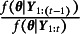 is difficult to evaluate, we can leverage the conditional independence assumption of the MIRT model. In particular, we can express the joint distribution of 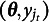 as 
(4.2)

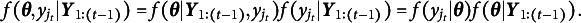

Using Equation (4.2), we rewrite the KL information term in Equation (3.3) as 

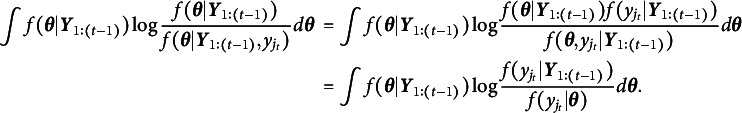

Since 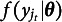 can be easily computed for each 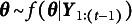 , the online computation of Max Pos remains efficient.

Although the MI selection rule in Equation (3.5) has demonstrated strong empirical performance (Wang & Chang, 2011), its computational complexity remains a significant challenge. By applying Equation (4.2), we can rewrite MI as 
(4.3)

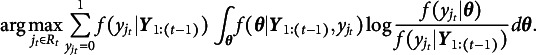

This formulation reveals that maximizing MI is structurally similar to Max Pos but requires much more computational effort. Unlike Max Pos, where sampling is performed from the current posterior 
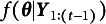
 , MI requires sampling from future posteriors 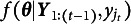 . Since each candidate item 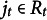 has two possible outcomes ( 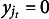 or 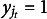 ), evaluating Equation (4.3) requires obtaining samples from 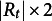 distinct posterior distributions. Even if sampling each individual posterior is computationally efficient, this approach becomes impractical for large item banks.

We hence propose a new approach to dramatically accelerate the computation of the MI quantity using the idea of importance sampling and resampling and bootstrap filter (Gordon et al., 1993; Smith & Gelfand, 1992). Rather than explicitly sampling the future posterior 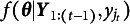 for each 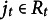 and 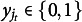 , we can simply sample from the current posterior 
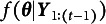
 once, and then perform proper posterior reweighting. Under Equation (3.1), let 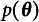 be the prior on the latent factors 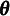 , 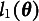 be the current data likelihood, and 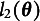 denote the future data likelihood after observing 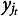 . We have the future posterior density as follows: 
(4.4)

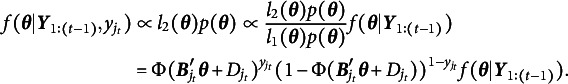

Equation (4.4) suggests that we can generate samples from 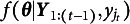 via reweighting and resampling from the current posterior samples 
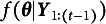
 . Specifically, given a sufficiently large set of posterior samples 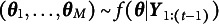 , we assign distinct weights for each sample 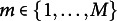 and future item 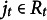 : 

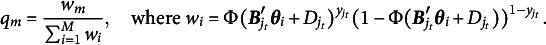

To sample from 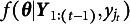 , we then draw from the discrete distribution over 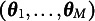 , placing weight 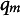 on 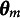 . This approach eliminates the need to sample from 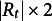 distinct posteriors directly, further accelerating MI computation and making it scalable for large item banks.

## 5 Learning optimal item-selection policy

Building on the Bayesian MIRT foundation established in the previous section, we now formulate the problem of CAT as an RL task. The Bayesian framework provides two key advantages that make this integration both principled and computationally feasible. First, the identified latent factor posterior distributions offer a well-defined representation of examinee knowledge, allowing their corresponding posterior parameters to be parameterized directly as state variables. Without such identification, encoding an unknown and analytically intractable posterior would be ambiguous. Second, because RL typically requires extensive simulations to learn an optimal policy, the acceleration achieved in online item selection enables rapid simulation of testing sessions, thereby substantially improving the efficiency of policy training.

As illustrated in Section 3.3, an RL approach addresses the myopic nature of traditional CAT selection rules, enables a more flexible reward structure, and directly minimizes the number of items required for performing online adaptive testing. Specifically, we propose a novel double Q-learning algorithm (Mnih et al., 2015; van Hasselt et al., 2016) for online item selection in CAT. The algorithm trains a deep neural network offline using only the item parameters estimated from an arbitrary two-parameter MIRT model. The offline training phase is completed before any live CAT administration. After training, the network weights are fixed, and online CAT only requires sequentially updating the posterior state and applying the learned network to select the next item. During online item selection, the neural network takes the current posterior distribution 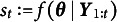 as input and outputs the next item selection for step 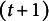 . The neural network architecture is described in Section 5.1, while the double deep Q-learning algorithm is detailed in Section 5.2.

### 5.1 Deep Q-learning neural network design

A potential reason that a deep RL approach has not been proposed in the CAT literature is the ambiguity arising from unidentified latent factor distributions. However, by Theorem 4.1, it is straightforward to compactly parameterize the posterior distribution at each time step *t* using the parameters 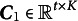 , 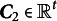 , and 
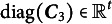
 , where 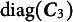 represents the diagonal vector of 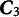 . This structured representation of the posterior enables item-selection policy learning via deep neural networks, which takes posterior parameters as input and outputs item selection.

Neural networks can be regarded as flexible function approximators that learn nonlinear mappings between inputs and outputs through a composition of simple transformations (Goodfellow et al., 2016). In our framework, the deep neural network consists of two key components: an encoder and a classifier. At each time step *t*, the encoder maps the collection of posterior parameters to a latent representation in 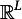 , where *L* is a hyperparameter and represents the dimension of the latent feature space. The classifier then outputs a *J*-dimensional vector of estimated Q-values and selects the next item from the item bank that is expected to yield the highest Q-value.

Define the collection of the posterior parameters 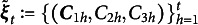 , where 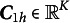 is the *h*th row of 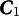 , 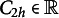 is the *h*th element of 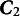 , and 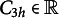 is the *h*th elements in the diagonal of 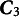 . Since the size of the posterior parameters 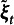 grows over time, and permuting the tuples within 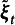 still describes the same posterior, we have to design a neural network that can take inputs of growing size, and can provide output that is permutation invariant of the inputs. One solution is to consider the weight sharing idea from the Bayesian experimental design literature (Foster et al., 2021). Let 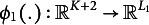 denote an encoder component that maps each tuple 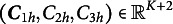 to an 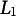 -dimensional latent space, and consider the operation 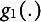 as follows: 
(5.1)

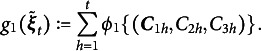

Observe that 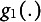 is capable of handling a growing number of inputs through summations of 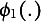 functions over *t*. More importantly, permuting the order of the tuples in 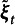 does not change the value of 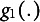 , since summation is permutation invariant. Although the form of 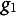 may look restrictive, any function 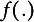 operating on a countable set can be decomposed into the form 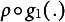 , where 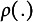 is a suitable transformation that can be learned from another neural network (see Theorem 2 of Zaheer et al., 2017).

In principle, the network design 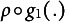 , coupled with the Q-learning algorithm, is sufficient to learn the optimal policy. However, to enhance learning efficiency, we further enrich the state representation 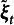 with a matrix of prediction quartiles 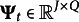 , where each row contains a vector of quantiles of the predictive distribution for item *j*. We form these quantiles by first drawing samples 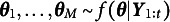 as in Section 4, and then computing the quantiles of the prediction samples 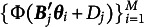 for each item *j*. Since sampling from 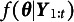 only needs to be done once, computing the matrix 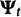 online is computationally efficient.

This practice of augmenting the raw state variable with additional contextual features of the states ( 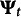 ) echoes the common strategy in the RL literature (Mnih et al., 2015; Schaul et al., 2015): learning tends to be more stable and efficient when the state representation captures not only the current ability estimate but also characteristics of the item bank. In our setting, 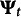 offers a richer description of the predictive distributions across items given the current estimates, effectively serving as additional covariates for item selection. Empirically, we find that incorporating 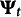 substantially accelerates convergence toward the optimal item-selection policy. Including 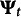 in the state representation is harmless, as it is a deterministic function of the posterior distribution; identical posteriors will always produce identical 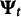 , ensuring that the policy remains consistent across equivalent states.

**Figure 2:**
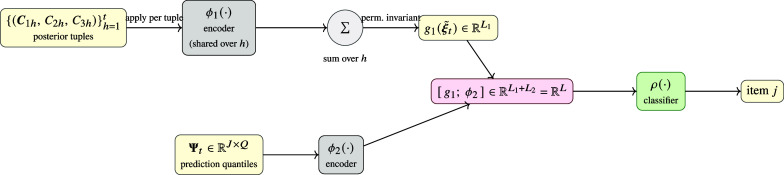
High-level architecture of the Q-network. The shared encoder 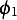 maps each tuple of posterior parameters to 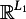 and the sum yields the permutation invariant representation 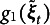 . The matrix 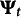 is encoded by 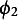 . The concatenated vector in 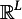 is passed to the classifier 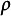 to select the *j*th item (largest value in the *J* logits). This network is trained offline using Algorithm 1; during live CAT, its weights are fixed and only the posterior state is updated sequentially.

In summary, Figure 2 describes the architecture of the policy network used in our approach. The same network is trained offline and then deployed during live CAT with fixed weights, so only the posterior state is updated sequentially during online administration. To select the 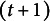 th item, our proposed neural net takes two inputs: the posterior parameters 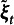 and the prediction matrix 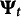 , and outputs the selected item. Let 
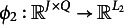
 represent the encoder component that maps the matrix 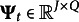 to 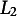 -dimensional space. Write 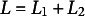 and the concatenated outputs of 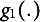 and 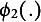 as 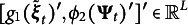 , we then define the classification component of neural network as 
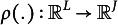
 , which maps the concatenated outputs of 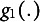 and 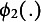 to a *J*-dimensional logit vector. Denote the final policy network as 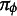 and recall that 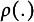 represents the classification layer, our proposed network selects the item at time *t* corresponding to the maximum value of the following function: 

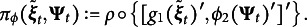

We implement both primary and target Q-networks as simple feed-forward multilayer perceptron and find that their performance is largely insensitive to the exact choice of 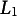 and 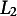 , and does not require very large depth. All architectural details, such as layer sizes, activations, and optimizer settings, are provided in Appendix D of the Supplementary Material. This robustness suggests that our empirical improvements stem from the CAT-specific state design and augmentation rather than network complexity.

### 5.2 Double deep Q-learning for CAT

In classical tabular Q-learning, the action-value function is stored as a Q-matrix whose rows correspond to states and whose columns correspond to actions. Such a representation is infeasible in CAT because the state space is combinatorially large and the state variable 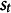 is represented by continuous posterior summaries rather than a small finite index. We therefore replace the tabular Q-matrix by a neural-network approximator 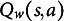 . For a given state 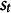 , the network in Figure 2 outputs the full vector of estimated Q-values before selecting the optimal item, which plays the same role as one row of a tabular Q-matrix, but is produced through function approximation rather than table lookup.

The standard Q-learning algorithm often overestimates Q-values because the same action-value approximation is used both to select the maximizing action and to evaluate it when constructing the temporal-difference target. To address this issue, we adopt the double Q-learning approach (van Hasselt et al., 2016), which replaces the single action-value approximation by two Q-networks with distinct roles. The primary network 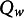 is updated by gradient descent and is used to select the action with the largest estimated Q-value, while the target network 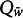 is a delayed copy used only to evaluate that selected action when forming the target. This is the neural-network analog of maintaining two Q-matrices in double Q-learning. The separation reduces overestimation bias and leads to more stable training. Specifically, compared with standard Q-learning, double Q-learning minimizes the same TD loss in (3.11) but replaces the target in (3.12) with 

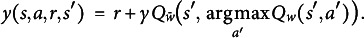



Our proposed algorithm is presented in Algorithm 1. The algorithm trains, entirely offline, the policy network illustrated in Figure 2. Once training is complete, the learned network is deployed during live CAT with fixed weights, and only the posterior state is updated sequentially as new responses are observed. Importantly, the offline training phase in Algorithm 1 relies only on the item parameters 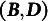 and simulated examinees drawn from a specified latent factor distribution (e.g., 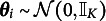 ), rather than on observed item response data from the item bank. If prior information about the distribution of future online examinees is available, the simulation distribution can be adapted accordingly. In this manuscript, we consistently use a standard multivariate normal distribution to ensure comparability across methods and experiments.

Note that for the Q-learning to converge to an optimal policy, it is essential to adopt an 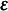 -greedy policy, where the algorithm makes random item selections with probability 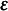 and gradually decreases 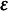 over the course of training. For simplicity, the state variable 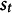 in Algorithm 1 is a compact representation of 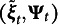 defined in Section 5.1. In practice, we can terminate the training when both the rewards and the validation loss stabilize, indicating that the neural network has well approximated the optimal policy.

**Figure:**
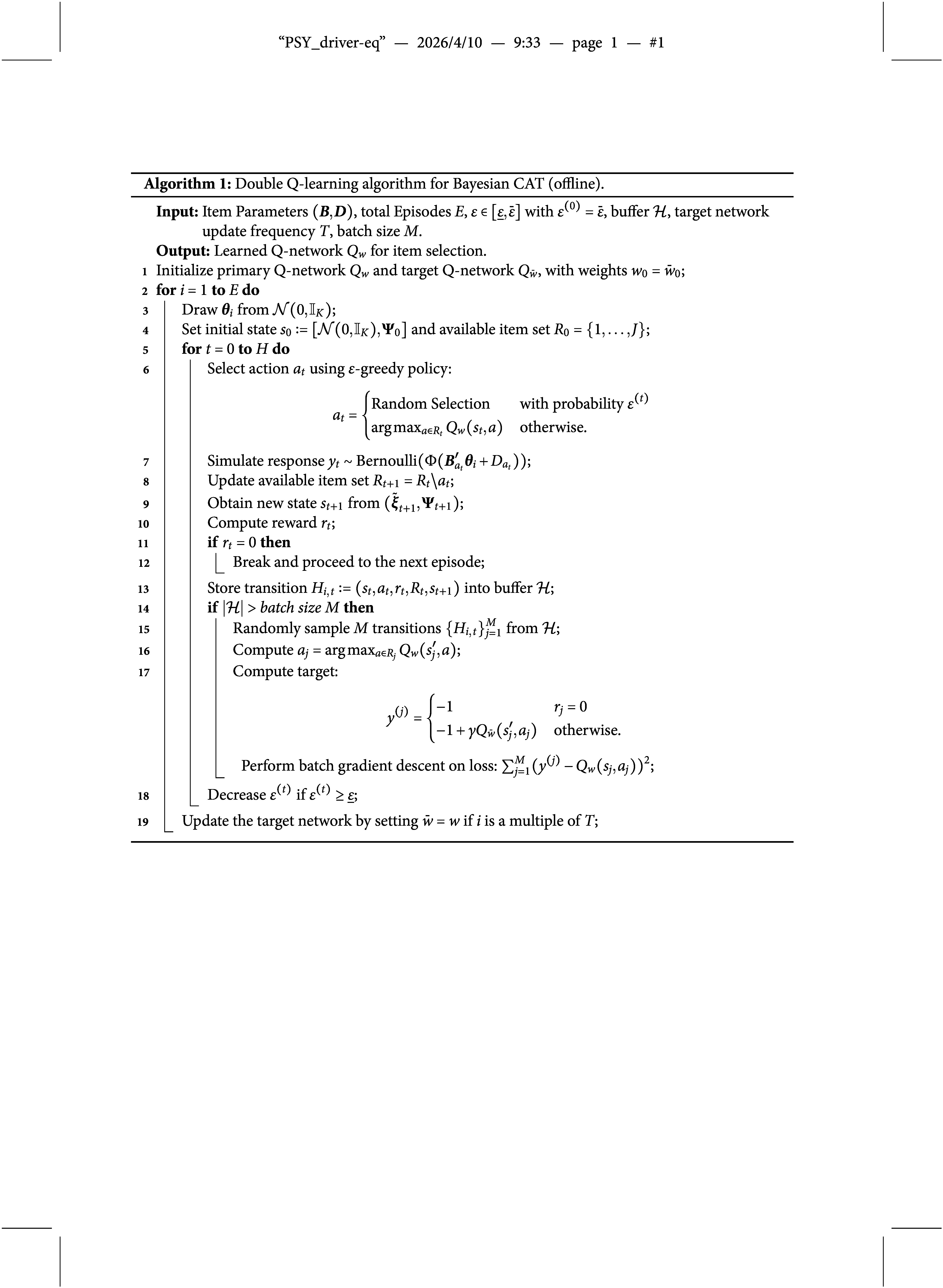

## 6 Simulation

### 6.1 Simulation design

To evaluate our approach in a challenging and realistic simulation setting, we randomly generated a 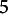 -factor, 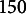 -item factor loading matrix 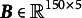 . Each column of 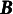 was initialized by randomly permuting 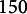 equally spaced values, with the magnitude constrained to lie within 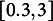 . We then imposed a lower triangular structure to ensure identifiability. To better reflect practical datasets, where items rarely load on all five factors, each item was required to load on the first factor and on at most two additional factors beyond the first. A visualization of the loading matrix can be found in Appendix F of the Supplementary Material. Item intercepts were independently drawn from 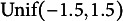 .

The goal of the simulation is to accurately estimate the first three factors ( 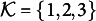 ), while accounting for the presence of factors 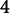 and 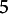 . Leveraging our proposed direct sampling approach outlined in Section 4, we implemented our double Q-learning algorithm alongside all existing methods discussed in Section 3.2. These include the EAP approach (Equation (3.2)), the Max Pos approach (Equation (3.3)), the MI approach (Equation (3.5)), and the Max Var approach (Equation (3.6)). We further modified all baseline information-based criteria so that they also target only the prioritized subset of factors 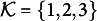 . Specifically, the EAP, Max Pos, and MI rules are now written as integrals over 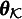 rather than all five factors. These adjustments ensure that all competing CAT algorithms are tuned to the same estimation target, making the simulation comparisons fair and directly comparable.

For performance evaluation, we generated 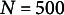 online examinees with latent traits 
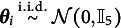
 . For each examinee, we administered a 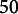 -item test using each of the CAT algorithms. We then compared performance in terms of posterior variance reduction, mean-squared error (MSE), termination efficiency, and item exposure rates based on these 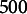 adaptive testing sessions.

For the Q-learning algorithm, we trained the Q-network for 80,000 episodes, with exploration parameter 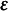 decreasing linearly from 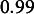 to 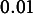 over 700,000 steps. We set a sufficiently large 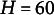 items, with discount factor 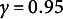 . To verify that the double Q-learning algorithm indeed converges to the optimal policy 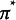 , we provide further details on the training dynamics of our deep Q-learning algorithm, illustrating the increase in rewards until convergence in Appendix F.2 of the Supplementary Material.

### 6.2 Simulation results

**Figure 3:**
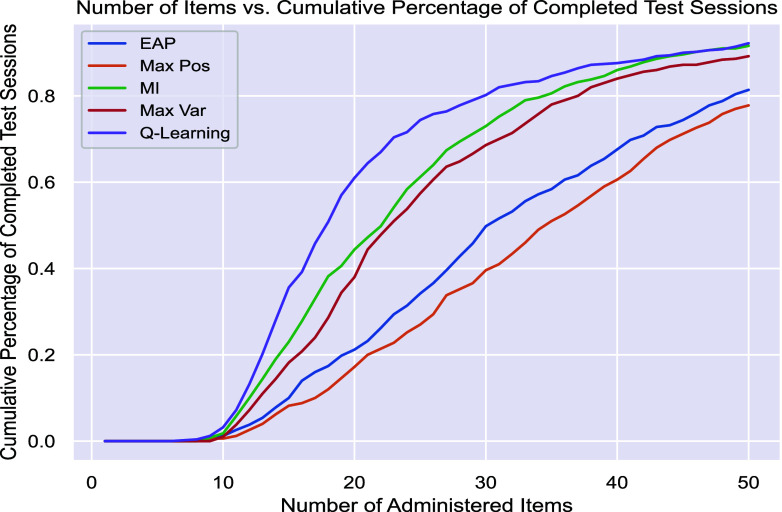
Number of items versus cumulative percentage of completed tests.

To evaluate termination efficiency, we start with a standard multivariate Gaussian prior with unit marginal variances and terminate the test once the maximum posterior variance across *all three* target factors in 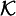 falls from 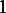 to below 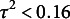 . In practice, practitioners may select the threshold 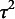 based on the specific requirements of their application. Based on 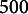 simulated adaptive testing sessions, Figure 3 illustrates how the percentage of completed test sessions increases as more items are administered. A faster growth rate of the completion percentages indicates faster posterior variance reduction. Notably, the purple Q-learning curve consistently rises more quickly than the others, demonstrating significant testing efficiency gains. As summarized in the second column of Table 1, Q-learning requires an average of only 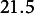 items to reach the termination criterion for all three targeted factors, outperforming all other methods.

**Table 1:** Comparison of win shares (W.S), termination, and computation

Algorithm	Avg termination (items)	W.S dim1	W.S dim2	W.S dim3	Avg time (s/item)
EAP	31.7	17.0%	16.4%	13.6%	**0.037**
Max Pos	34.0	16.8%	16.2%	18.6%	0.055
MI	23.2	19.0%	21.4%	20.2%	0.082
Max Var	25.7	23.6%	22.6%	23.6%	0.038
Q-learning	**21.5**	**23.6**%	**23.4%**	**24.0%**	0.064

To assess early-stage performance, we compute the MSEs after 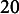 items for each test session and summarize results using win shares, defined as the percentage of test takers for whom each method achieves the lowest MSE after 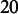 items. These win shares are reported across all three latent dimensions in Table 1. Notably, Q-learning achieves the best win shares across all three factors. This advantage at early stages is consistent with the training objective of Q-learning, which explicitly rewards rapid posterior variance reduction and early test termination, with the average stopping time occurring at around 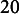 items.

Table 2 reports the evolution of MSEs as a function of test length up to 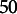 administered items. Consistent with the win share analysis, Q-learning exhibits competitive performance during the early stages of testing. In particular, at 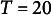 and 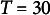 , Q-learning attains the smallest MSEs for Factors 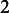 and 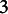 . As the test length increases, performance differences across methods diminish, and Q-learning achieves MSEs comparable to those of MI and Max Var. This pattern is expected, as the Q-learning policy is trained primarily on short-horizon testing trajectories: since most simulated tests terminate before 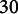 items, the algorithm is exposed less frequently to long-horizon simulations during training. Additional visualizations of the MSE trajectories are provided in Appendix F.3 of the Supplementary Material.

**Table 2:** MSEs between posterior mean and ground truth for the first three latent factors as a function of test length

		Number of items				
Factor	Algorithm	10	20	30	40	50
1	EAP	0.175	0.123	0.090	0.073	0.062
	Max Pos	0.194	0.128	0.104	0.083	0.062
	MI	**0.180**	0.100	**0.066**	**0.055**	**0.050**
	Max Var	0.186	**0.080**	0.073	0.059	0.056
	Q-learning	0.221	0.096	0.071	0.060	0.054
2	EAP	0.362	0.214	0.164	0.125	0.105
	Max Pos	0.354	0.221	0.166	0.129	0.103
	MI	0.223	0.150	0.120	0.102	**0.086**
	Max Var	**0.214**	0.141	0.118	**0.092**	0.087
	Q-learning	0.282	**0.138**	**0.111**	0.096	0.087
3	EAP	0.317	0.226	0.152	0.112	0.085
	Max Pos	0.324	0.207	0.139	0.115	0.092
	MI	**0.213**	0.127	0.103	0.096	0.089
	Max Var	0.234	0.136	0.110	0.099	0.096
	Q-learning	0.233	**0.114**	**0.098**	**0.091**	**0.085**

**Figure 4:**
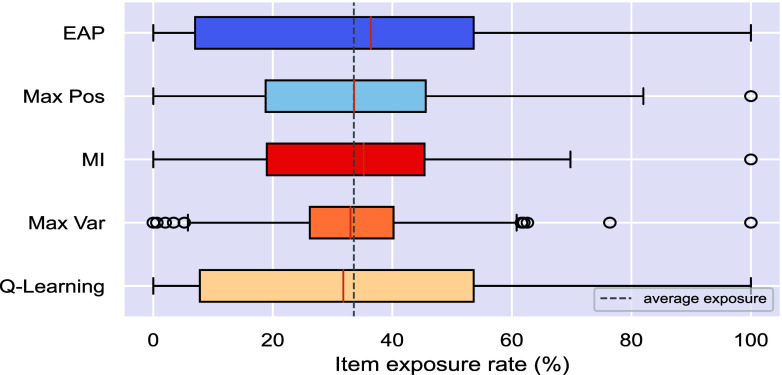
Distributions of item exposure rates.

Figure 4 summarizes the distribution of item exposure rates across the 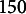 item bank for each CAT algorithm, where the exposure rate of an item is defined as the proportion of examinees whose test includes that item at least once. With test length 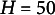 and bank size 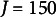 , the expected average exposure is 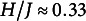 , and all five algorithms yield mean and median exposure rates close to this benchmark. Differences across methods primarily arise in the tails of the exposure distribution. In particular, the EAP and Q-learning policies exhibit slightly higher upper quartile and maximum exposure rates, indicating a more selective use of highly informative items. Importantly, no method concentrates exposure excessively on a small subset of items, suggesting that all algorithms maintain reasonable item utilization in this simulation setting. For the Q-learning approach, incorporating explicit exposure control mechanisms into the learning objective, such as penalizing repeated use of highly exposed items, is a natural extension but is beyond the scope of the present study.

The last column of Table 1 reports the average online item-selection time, highlighting the significant computational advantages of our direct sampling approach. Even the most computationally intensive MI approach requires only 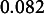 seconds per item selection, via our proposed posterior reweighting strategy. Q-learning adds only a single feed-forward pass per selection, keeping online latency under a few milliseconds on standard hardware without GPU acceleration. While training the Q-network offline using Algorithm 1 took approximately 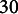 hours for this exercise, this is a one-time investment that can be accelerated if GPUs are available; thereafter, the virtually instantaneous online policy makes the approach highly practical for real-time CAT deployment.

## 7 Cognitive function measurements

### 7.1 pCAT-COG data and experiment design

We revisit the problem of designing a deep CAT system for the pCAT-COG study, as outlined in Section 2. Since item response data for all 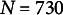 examinees across 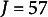 items are available, we can directly use real item responses during evaluation rather than simulating testing sessions. In this experiment, all 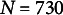 examinees are treated as online examinees. All CAT algorithms, including Q-learning, have access only to the estimated item parameters from the pCAT-COG study and do not observe the examinees’ binary item responses beyond those revealed sequentially during adaptive administration.

Given that pCAT-COG is designed to measure global cognitive ability (first column in Figure 1) while accounting for five cognitive subdomains, we specified the Q-learning reward function to reduce posterior variance for the primary factor ( 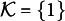 ). This demonstrates the flexibility of our CAT system, as it can be tailored to the specific cognitive assessment needs. As in Section 6, we also modified all baseline CAT algorithms so that they also target only the primary factor by integrating only over the primary dimension for fair comparison.

Finally, this real-data experiment also serves as a robustness check for the proposed Q-learning approach. As described in Algorithm 1, the policy is trained using simulated examinees with latent factors drawn from a standard multivariate normal distribution. In contrast, the empirical factor correlations in pCAT-COG need not follow this distributional assumption. Evaluating Q-learning on real response data therefore provides evidence that the learned policy remains effective when the latent factor structure deviates from the training distribution used in simulation.

### 7.2 Results

**Figure 5:**
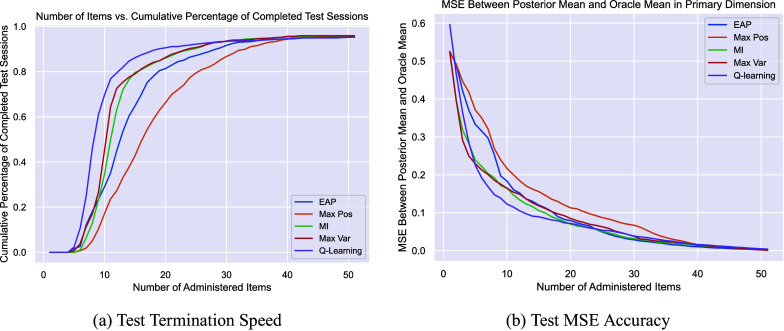
pCAT-COG: Primary factor posterior variance reduction (left) and estimation accuracy (right).

The left subplot of Figure 5 shows that Q-learning again achieves the fastest test termination compared to other methods. As before, the test is dynamically terminated when the posterior variance drops from 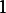 to below 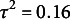 , and the cumulative percentage of completed test sessions is computed over all 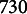 examinees. The second column of Table 3 shows that Q-learning reaches the desired posterior variance reduction threshold after only an average of 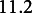 items.

**Table 3:** Comparison of termination efficiency, primary-factor accuracy, and computation for pCAT-COG

Algorithm	Avg termination (items)	Correlation	Win shares	Avg time (s/item)
EAP	15.1	0.956	17.7%	**0.020**
Max Pos	18.5	0.933	16.7%	0.030
MI	12.6	0.959	21.8%	0.038
Max Var	13.0	0.951	20.9%	0.021
Q-learning	**11.2**	**0.959**	**22.9**%	0.025

*Note*: Primary factor correlation and win shares (W.S) are computed after 20 administered items. Win shares denote the proportion of examinees for whom a method attains the smallest mean-squared error for the primary factor at this stage.

For further comparison, we consider adaptively selecting 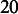 items for each test taker without dynamic termination. We chose the number 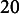 because the Max Pos approach required the largest average of 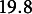 items for termination. The third column of Table 3 shows that the Q-learning approach achieves the highest 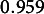 correlation between the estimated primary factor posterior means and the ground truth after only 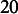 items. Additionally, the Q-learning approach attains the highest win shares in estimating the primary dimension across all 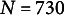 examinees after 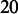 items. As defined in Section 6, win shares denote the proportion of examinees for whom a CAT method attains the smallest MSE. Since all 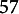 items are ultimately administered in the real-data study, exposure rates are degenerate under full-length testing. We therefore report item exposure patterns based on the first 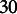 administered items in Appendix G.1 of the Supplementary Material.

Additionally, the right subplot of Figure 5 highlights the rapid decay of MSE in estimating the primary dimension with Q-learning. As summarized in Table 4, Q-learning achieves the smallest MSE at the early stages when 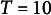 and 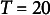 . At longer horizons when 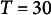 and 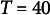 , the gains in MSE for the Q-learning approach diminish. This behavior is consistent with the simulation findings in Section 6 and reflects the reward specification used for Q-learning, which emphasizes rapid early-stage variance reduction. Because most simulated training trajectories generated in Algorithm 1 terminate well before 20 items, such performance behavior for Q-learning is expected.

**Table 4:** Mean-squared errors (MSEs) as a function of test length

Algorithm	10 items	20 items	30 items	40 items
EAP	0.183	0.079	**0.029**	**0.010**
Max Pos	0.217	0.113	0.067	0.016
MI	0.165	**0.070**	0.032	0.014
Max Var	0.165	0.085	0.038	0.014
Q-learning	**0.123**	0.072	0.038	0.016

This experiment highlights the effectiveness of our Q-learning approach in high-dimensional cognitive function measurement. Unlike approaches that learn a fixed test design offline (Krantsevich et al., 2023), our framework enables fully dynamic item selection, maximizing the utilization of the item bank by exploring diverse testing trajectories. This termination efficiency is particularly valuable in cognitive assessment, where item development is costly, and can help mitigate practice effects and preserve items for future use.

## 8 Discussion

This work advocates for a deep RL perspective in the design of MCAT systems. We make two key contributions to the existing CAT literature: (1) a computational advancement that accelerates existing online item-selection rules within a flexible Bayesian MIRT framework and (2) a novel RL-based approach that mitigates myopic decision-making and prioritizes the assessment of primary factors of interest.

Theorem 4.1 not only provides an efficient parameterization of the latent factor posterior distributions for our proposed deep Q-learning approach, but also improves existing CAT item-selection algorithms, as detailed in Section 4. Leveraging direct sampling from unified skew-normal distributions, our methodology scales efficiently with a large number of factors and items, achieving near-instantaneous online selection by circumventing MCMC sampling and data augmentation. Additionally, our approach naturally extends to fully Bayesian item selection by accounting for uncertainties in item parameters, which is an essential consideration when the item bank is not well-calibrated as detailed in Appendix C of the Supplementary Material. Throughout the testing trajectory, our approach precisely characterizes the evolution of posterior distributions at each time step, providing a more robust measurement process beyond point estimates with difficult-to-compute standard errors.

Another key contribution of this work is the development of a robust deep double Q-learning algorithm with a customized reward structure that directly minimizes test length. As demonstrated in both simulations and real-data studies, our Q-learning algorithm consistently achieves the fastest posterior variance reduction while rapidly decreasing estimation bias. Moreover, its flexible reward function allows adaptation to different testing objectives, providing a principled framework for designing customized tests and overcoming the myopic nature of traditional CAT methods.

Our work also offers practical guidance for selecting the appropriate item-selection algorithms. Unlike the existing heuristic rules, one limitation of our deep CAT system is the requirement of offline training (Algorithm 1) before online deployment. For the pCAT-COG study, offline training took approximately 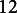 hours on a single GPU as a one-time investment, but no GPU is needed for subsequent online item selection. Even when offline training is undesirable, our framework significantly accelerates existing methods. Experiments show that MI (Equation (3.5)) and our Max Var (Equation (3.6)) approaches often outperform other online item-selection rules, echoing the findings presented in Wang and Chang (2011).

A promising direction for future work is to address several limitations of the current Q-learning framework for CAT. First, the learned policy is tied to a specific item bank and must be retrained when the items are substantially modified or replenished, which may limit its immediate applicability in settings with frequent item updates. Second, as a model-free approach, Q-learning can be computationally expensive to train, particularly for long-horizon testing scenarios in which optimal policies depend on extended future trajectories. In addition, although the proposed approach does not rely on tabular exploration of the full state space and instead uses function approximation based on a compact posterior representation, scalability remains an important practical consideration as the state space grows exponentially. Our simulation results suggest that the method works well for moderate 150-item banks, but additional methodological development may be useful for extending the approach to substantially larger banks. Finally, while the proposed method yields reasonable item exposure patterns in our experiments, additional work is needed to incorporate explicit exposure control mechanisms directly into the learning objective.

Another exciting avenue is to explore alternative reward structures for our Q-learning algorithm. While the 0–1 reward structure is interpretable and stabilizes Q-network training, it provides sparse feedback, which may limit empirical performance. It is also important to understand when RL truly improves on myopic rules, since Chan and Farias (2009) showed that greedy policies can be near-optimal in some dynamic stochastic optimization problems. This requires careful assumptions about the reward function and the item bank properties, providing deeper insights into the trade-offs between RL and traditional item-selection strategies.

## Supporting information

10.1017/psy.2026.10106.sm001 Li et al. supplementary material Li et al. supplementary material

## Data Availability

The data and code that support the findings of this study are publicly available at the GitHub repository: https://github.com/JiguangLi/deep_CAT/tree/deep_cat.

## Supplementary material

The supplementary material for this article can be found at https://doi.org/10.1017/psy.2026.10106.
