# Supplementary material for: Deep Computerized Adaptive Testing
Source: Psychometrika. 2026 Mar 27:1–22. Online ahead of print. doi: 10.1017/psy.2026.10106 (PMC13274932; doi:10.1017/psy.2026.10106)
Supplement: Li et al. supplementary material [file S0033312326101069sup001.pdf]

Supplementary Material: Deep Computerized Adaptive Testing

A. Mutual Information as Prediction Uncertainties

We observe that all the heuristic Bayesian item selection methods discussed in this manuscript account for prediction uncertainty. In particular, the mutual information item selection rule can be rewritten as follows:

$$
\begin{aligned}
\arg\max_{j_t\in R_t} I_M(\boldsymbol{\theta}, y_{j_t}) &= \arg\max_{j_t\in R_t} \sum_{y_{j_t}=0}^{1} \int_{\boldsymbol{\theta}} f(\boldsymbol{\theta}, y_{j_t}|\boldsymbol{Y}_{1:(t-1)}) \log \frac{f(\boldsymbol{\theta}, y_{j_t}|\boldsymbol{Y}_{1:(t-1)})}{f(\boldsymbol{\theta}|\boldsymbol{Y}_{1:(t-1)})f(y_{j_t}|\boldsymbol{Y}_{1:(t-1)})} d\boldsymbol{\theta} \\
&= \arg\max_{j_t\in R_t} \sum_{y_{j_t}=0}^{1} \int_{\boldsymbol{\theta}} f(y_{j_t}|\boldsymbol{\theta}) f(\boldsymbol{\theta}|\boldsymbol{Y}_{1:(t-1)}) \log \frac{f(\boldsymbol{\theta}, y_{j_t}|\boldsymbol{Y}_{1:(t-1)})}{f(\boldsymbol{\theta}|\boldsymbol{Y}_{1:(t-1)})f(y_{j_t}|\boldsymbol{Y}_{1:(t-1)})} d\boldsymbol{\theta} \\
&= \arg\max_{j_t\in R_t} \sum_{y_{j_t}=0}^{1} \int_{\boldsymbol{\theta}} f(y_{j_t}|\boldsymbol{\theta}) f(\boldsymbol{\theta}|\boldsymbol{Y}_{1:(t-1)}) \log \frac{f(y_{j_t}|\boldsymbol{\theta})}{f(y_{j_t}|\boldsymbol{Y}_{1:(t-1)})} d\boldsymbol{\theta} \\
&= \arg\max_{j_t\in R_t} \int_{\boldsymbol{\theta}} [\Phi(\boldsymbol{B}'_{j_t}\boldsymbol{\theta} + D_{j_t}) \log \frac{\Phi(\boldsymbol{B}'_{j_t}\boldsymbol{\theta} + D_{j_t})}{c_{j_t}} + \qquad \text{(A.1)} \\
&\qquad (1 - \Phi(\boldsymbol{B}'_{j_t}\boldsymbol{\theta} + D_{j_t})) \log \frac{(1 - \Phi(\boldsymbol{B}'_{j_t}\boldsymbol{\theta} + D_{j_t}))}{(1 - c_{j_t})}] f(\boldsymbol{\theta}|\boldsymbol{Y}_{1:(t-1)}) d\boldsymbol{\theta}
\end{aligned}
$$

Equation (A.1) suggests the mutual information criterion selects the item j_t that has the largest expected KL divergence between the Bernoulli distributions parametrized by $\Phi(\boldsymbol{B}'_{j_t}\boldsymbol{\theta} + D_{j_t})$ and c_{j_t}, weighted by the current posterior $f(\boldsymbol{\theta}|\boldsymbol{Y}_{1:(t-1)})$.

Observe that mutual information essentially favors the item j_t that has the largest prediction uncertainties around its prediction mean c_{j_t}, quantified by the KL divergence. To compare equation (A.1) with equation (3.6), note that we have replace the integral of KL divergence from c_{j_t} with variances $(\Phi(\boldsymbol{B}'_{j_t}\boldsymbol{\theta} + D_{j_t}) - c_{j_t})^2$ in (3.6). This suggests that our proposed approach shares similar theoretical properties with the mutual information criterion, but offers a much simpler formula for online computation, avoiding posterior reweighting and log transformations. Empirically, our proposed

item selection rule achieves comparable performance to mutual information but with significantly reduced computational time.

B. Sequential Sampling in CAT

B.1. Proof of Theorem 4.1

For any $T \geq 1$, we may write the likelihood of item response after administering T items under the two-parameter probit MIRT model as follows:

$$\prod_{t=1}^{T} \Phi(\boldsymbol{B}'_{j_t}\boldsymbol{\theta} + D_{j_t})^{y_{j_t}}(1 - \Phi(\boldsymbol{B}'_{j_t}\boldsymbol{\theta} + D_{j_t}))^{1-y_{j_t}} = \prod_{t=1}^{T} \Phi\{(2y_{j_t} - 1)(\boldsymbol{B}'_{j_t}\boldsymbol{\theta} + D_{j_t})\}$$

$$= \Phi_T(\boldsymbol{C}_1\boldsymbol{\theta} + \boldsymbol{C}_2;\ \mathbb{I}_T)$$

Given the standard multivariate normal prior $\boldsymbol{\theta} \sim N(0, \mathbb{I}_K)$, the posterior can be expressed as follows:

$$\begin{aligned}\pi(\boldsymbol{\theta}|\boldsymbol{Y}_{1:T}, \boldsymbol{B}_{1:T}, \boldsymbol{D}_{1:T}) &\propto \phi_K(\boldsymbol{\theta}; \boldsymbol{0}, \mathbb{I}_K)\Phi_T\{(\boldsymbol{C}_1\boldsymbol{\theta} + \boldsymbol{C}_2); \mathbb{I}_T\} \\ &= \phi_K(\boldsymbol{\theta}; \boldsymbol{0}, \mathbb{I}_K)\Phi_T\{\boldsymbol{C}_3^{-1}(\boldsymbol{C}_1\boldsymbol{\theta} + \boldsymbol{C}_2); \boldsymbol{C}_3^{-1}\boldsymbol{C}_3^{-1}\} \\ &= \phi_K(\boldsymbol{\theta}; \boldsymbol{0}, \mathbb{I}_K)\Phi_T\{\boldsymbol{C}_3^{-1}\boldsymbol{C}_2 + \boldsymbol{C}_3^{-1}\boldsymbol{C}_1\boldsymbol{\theta}; \boldsymbol{C}_3^{-1}\boldsymbol{C}_3^{-1}\}.\end{aligned}$$

To identify the exact posterior parameters, we draw back to our attention to the probability kernel of the unified skew-normal distribution as defined in Definition 4.1. We observe that the posterior density above aligns with the SUN density. Specifically, if $\boldsymbol{\theta} \sim \text{SUN}_{K,T}(\boldsymbol{\mu}, \boldsymbol{\Omega}, \boldsymbol{\Delta}, \boldsymbol{\gamma}, \boldsymbol{\Gamma})$, its density is

$$\phi_K\big(\boldsymbol{\theta}; \boldsymbol{\mu}, \boldsymbol{\Omega}\big)\ \frac{\Phi_T\Big(\boldsymbol{\gamma} + \boldsymbol{\Delta}^\top\bar{\boldsymbol{\Omega}}^{-1}\boldsymbol{\Omega}^{-1}(\boldsymbol{\theta} - \boldsymbol{\mu})\ ;\ \boldsymbol{\Gamma} - \boldsymbol{\Delta}^\top\bar{\boldsymbol{\Omega}}^{-1}\boldsymbol{\Delta}\Big)}{\Phi_T(\boldsymbol{\gamma}; \boldsymbol{\Gamma})}.$$

We can interpret this as a product of a K-variate Gaussian pdf in $\boldsymbol{\theta}$ and a T-variate Gaussian cdf in $\boldsymbol{\theta}$, with normalizing constant $\Phi_T(\boldsymbol{\gamma}; \boldsymbol{\Gamma})$, since $\Phi_T(\boldsymbol{\gamma}; \boldsymbol{\Gamma})$ is not a function of $\boldsymbol{\theta}$. But this exactly matches our derived latent-factor posterior $\pi(\boldsymbol{\theta} \mid$

$\boldsymbol{Y}_{1:T}, \boldsymbol{B}_{1:T}, \boldsymbol{D}_{1:T}$) under the MIRT model. It remains to identify $(\boldsymbol{\mu}, \boldsymbol{\Omega}, \boldsymbol{\Delta}, \boldsymbol{\gamma}, \boldsymbol{\Gamma})$ by matching $\phi_K(\boldsymbol{\theta}; \boldsymbol{\mu}, \boldsymbol{\Omega})\Phi_T\left(\boldsymbol{\gamma} + \boldsymbol{\Delta}^\top\bar{\boldsymbol{\Omega}}^{-1}\boldsymbol{\Omega}^{-1}(\boldsymbol{\theta} - \boldsymbol{\mu})\,;\, \boldsymbol{\Gamma} - \boldsymbol{\Delta}^\top\bar{\boldsymbol{\Omega}}^{-1}\boldsymbol{\Delta}\right)$ to

$$\phi_K(\boldsymbol{\theta}; \mathbf{0}, \mathbf{I}_K)\, \Phi_T\left(\mathbf{C}_3^{-1}\mathbf{C}_2 + \mathbf{C}_3^{-1}\mathbf{C}_1\boldsymbol{\theta}\,;\, \mathbf{C}_3^{-1}(\mathbf{C}_3^{-1})^\top\right).$$

Matching the pdf part immediately yields $\boldsymbol{\mu}_{\text{post}} = 0_K$ and $\boldsymbol{\Omega}_{\text{post}} = \mathbb{I}_K$. To match the cdf part, we need to match $\boldsymbol{\gamma} + \boldsymbol{\Delta}^\top\bar{\boldsymbol{\Omega}}^{-1}\boldsymbol{\Omega}^{-1}(\boldsymbol{\theta} - \boldsymbol{\mu})$ to $\mathbf{C}_3^{-1}\mathbf{C}_2 + \mathbf{C}_3^{-1}\mathbf{C}_1\boldsymbol{\theta}$, which implies $\boldsymbol{\gamma}_{\text{post}} = \boldsymbol{C}_3^{-1}\boldsymbol{C}_2$.

It remains to solve the following two linear equations:

$$\boldsymbol{\Delta}'\bar{\boldsymbol{\Omega}}^{-1}\boldsymbol{\Omega}^{-1} = \boldsymbol{C}_3^{-1}\boldsymbol{C}_1$$

$$\boldsymbol{\Gamma} - \boldsymbol{\Delta}'\bar{\boldsymbol{\Omega}}^{-1}\boldsymbol{\Delta} = \boldsymbol{C}_3^{-1}\boldsymbol{C}_3^{-1}.$$

Recall that in definition 4.1, we let $\boldsymbol{\Omega} = \boldsymbol{\omega}\bar{\boldsymbol{\Omega}}\boldsymbol{\omega}$ be the decomposition of covariance matrix into correlation matrix. Since $\boldsymbol{\Omega}_{\text{post}} = \mathbb{I}_K$, we have $\bar{\boldsymbol{\Omega}} = \boldsymbol{\Omega} = \mathbb{I}_K$. It follows that solving the first equation yields $\boldsymbol{\Delta}_{\text{post}} = \boldsymbol{C}_1'\boldsymbol{C}_3^{-1}$. Plugging our solution of $\boldsymbol{\Delta}_{\text{post}}$ in the second solution yields

$$\boldsymbol{\Gamma}_{\text{post}} = \boldsymbol{C}_3^{-1}\boldsymbol{C}_3^{-1} + \boldsymbol{C}_3^{-1}\boldsymbol{C}_1\boldsymbol{C}_1'\boldsymbol{C}_3^{-1} = \boldsymbol{C}_3^{-1}(\boldsymbol{C}_1\boldsymbol{C}_1' + \mathbb{I}_T)\boldsymbol{C}_3^{-1}.$$

Finally, to show this is indeed a unified skew-normal distribution, we need to show the matrix $\boldsymbol{\Omega}^*$ as defined in Definition 4.1 is indeed a full-rank correlation matrix. To see this, we may decompose $\boldsymbol{\Omega}^*$ as follows:

$$\begin{bmatrix} \boldsymbol{C}_3^{-1}(\boldsymbol{C}_1\boldsymbol{C}_1' + \mathbb{I}_T)\boldsymbol{C}_3^{-1} & \boldsymbol{C}_3^{-1}\boldsymbol{C}_1 \\ \boldsymbol{C}_1'\boldsymbol{C}_3^{-1} & \mathbb{I}_K \end{bmatrix} = \begin{bmatrix} \boldsymbol{C}_3^{-1} & 0 \\ 0 & \mathbb{I}_K \end{bmatrix} \times \begin{bmatrix} \boldsymbol{C}_1\boldsymbol{C}_1' + \mathbb{I}_T & \boldsymbol{C}_1 \\ \boldsymbol{C}_1' & \mathbb{I}_K \end{bmatrix} \times \begin{bmatrix} \boldsymbol{C}_3^{-1} & 0 \\ 0 & \mathbb{I}_K \end{bmatrix}.$$

Observe that this is a decomposition of a correlation matrix, where the middle matrix above is the covariance matrix of a $(T+K)$ dimensional random vector $[z_1', z_2']'$, where

$z_1 = \boldsymbol{C}_1 z_2 + \boldsymbol{\epsilon}$, z_2 is a K-dimensional standard multivariate Gaussian vector with identity covariance matrix, and $\boldsymbol{\epsilon}$ is a T-dimensional standard multivariate Gaussian vector, independent of z_2.

B.2. Sequential Sampling Algorithms

For any Bayesian item selection algorithms, including our proposed deep CAT framework, it is essential to sample from the latent factor posterior distributions $f(\boldsymbol{\theta}|\boldsymbol{Y}_{1:T})$ for any $T \geq 1$. Theorem 4.1 provides a direct sampling approach to perform such sequential sampling, hence circumventing the need for MCMC algorithms, which cannot be parallelized and require additional tuning and mixing time.

The key observation is that any unified skew-normal distribution $\boldsymbol{\theta} \sim \mathrm{SUN}_{K,T}(\boldsymbol{\mu}, \boldsymbol{\Omega}, \boldsymbol{\Delta}, \boldsymbol{\gamma}, \boldsymbol{\Gamma})$ has the stochastic representation as $\boldsymbol{\theta} \overset{d}{=} \boldsymbol{\mu} + \omega(\boldsymbol{V}_0 + \boldsymbol{\Delta}\boldsymbol{\Gamma}^{-1}\boldsymbol{V}_{1,-\gamma})$, where $\boldsymbol{V}_0 \sim N(0, \bar{\boldsymbol{\Omega}} - \boldsymbol{\Delta}\boldsymbol{\Gamma}^{-1}\boldsymbol{\Delta}') \in \mathbb{R}^K$ and $V_{1,-\gamma}$ is obtained by component-wise truncation below $-\boldsymbol{\gamma}$ of a variate $N(0, \boldsymbol{\Gamma}) \in \mathbb{R}^T$. By plugging in the posterior parameters derived in Theorem 4.1, we have the representation

$$\boldsymbol{\theta} \sim \boldsymbol{V}_0 + \boldsymbol{C}_1'(\boldsymbol{C}_1\boldsymbol{C}_1' + \mathbb{I}_T)^{-1}\boldsymbol{C}_3\boldsymbol{V}_{1,-\gamma}.$$

Hence we can conduct efficient sequential posterior sampling using the following three steps when $t > 1$:

- **Step One**: Sample from multivariate normal distribution

$$\boldsymbol{V}_0 \sim N\Big(0, \mathbb{I}_K - \boldsymbol{C}_1'(\boldsymbol{C}_1\boldsymbol{C}_1' + \mathbb{I}_T)^{-1}\boldsymbol{C}_1\Big).$$

- **Step Two**: Leveraging the minimax tilting method Botev, 2016, sample from the zero-mean T-variate truncated multivariate normal distribution $\boldsymbol{V}_{1,-\gamma}$ with covariance matrix $\boldsymbol{C}_3^{-1}(\boldsymbol{C}_1\boldsymbol{C}_1' + \mathbb{I}_T)\boldsymbol{C}_3^{-1}$ and truncation below -$\boldsymbol{C}_3^{-1}\boldsymbol{C}_2$.

- **Step Three**: perform linear computation $\boldsymbol{V}_0 + \boldsymbol{C}_1'(\boldsymbol{C}_1\boldsymbol{C}_1' + \mathbb{I}_T)^{-1}\boldsymbol{C}_3 V_{1,-\gamma}$.

Note for $t = 1$, the sampling steps above can be modified into their univariate analogue. Since steps one and step two are independent, one can simply perform i.i.d sampling from both $\boldsymbol{V}_0$ and $\boldsymbol{V}_{1,-\gamma}$ in parallel to draw large samples of $\boldsymbol{\theta} \sim f(\boldsymbol{\theta}|\boldsymbol{Y}_{1:T})$ in the probit MIRT model. The $t \times t$ matrix inverse $(\boldsymbol{C}_1\boldsymbol{C}_1' + \mathbb{I}_T)$ is easy to compute and only needs to be computed once.

C. Fully Bayesian Item Selection

C.1. Generalizing to Fully Bayesian Item Selection

Another key advantage of our direct sampling approach is its ability to enable rapid fully Bayesian item selection. Since Section 3, we have assumed that the item bank is well-calibrated, allowing the factor loading matrix and the intercept to be treated as fixed. According to van der Linden and Ren (2020), this assumption can lead to overly optimistic estimation of the latent traits in practice, and is only reasonable when large calibration datasets are available. A fully Bayesian framework offers a principled way to incorporate this uncertainty by integrating over the joint posterior of item parameters and latent traits. However, performing this integration in online item selection has long been considered intractable, limiting its practical adoption. Notably, van der Linden and Ren (2020) introduced an efficient MCMC method, but only for unidimensional item response theory model.

Our direct sampling approach extends fully Bayesian item selection to multidimensional IRT while avoiding the computational bottlenecks of MCMC. To illustrate, we consider the mutual information criterion in (4.3). Fully Bayesian inference involves obtaining posterior samples of item parameters $\{\boldsymbol{\xi}^{(m)}\}_{m=1}^{M}$, where $\boldsymbol{\xi}^{(m)} := (\boldsymbol{B}^{(m)}, \boldsymbol{D}^{(m)})$. The mutual information criterion in Equation (4.3) requires computing

the posterior predictive probability:

$$f(y_{j_t}|\boldsymbol{Y}_{1:(t-1)}) = \int_{\boldsymbol{\xi}}\int_{\boldsymbol{\theta}} f(y_{j_t}|\boldsymbol{\theta}) f(\boldsymbol{\theta}|\boldsymbol{\xi}, \boldsymbol{Y}_{1:(t-1)}) d\boldsymbol{\theta} d\boldsymbol{\xi}. \tag{C.1}$$

Similarly, the KL divergence term must integrate out the nuisance item parameters $\boldsymbol{\xi}$:

$$\int_{\boldsymbol{\xi}}\int_{\boldsymbol{\theta}} f(\boldsymbol{\theta}|\boldsymbol{Y}_{1:(t-1)}, y_{j_t}, \boldsymbol{\xi}) \log \frac{f(y_{j_t}|\boldsymbol{\theta})}{f(y_{j_t}|\boldsymbol{Y}_{1:(t-1)})} d\boldsymbol{\theta} d\boldsymbol{\xi}. \tag{C.2}$$

By leveraging Theorem 4.1, we can exactly identify the posterior distributions for any arbitrary configuration of $f(\boldsymbol{\theta}|\boldsymbol{Y}_{1:(t-1)}, y_{j_t}, \boldsymbol{\xi}^{(m)})$, hence enabling direct sampling in parallel. It follows Equations (C.1) and (C.2) are computationally feasible via Monte Carlo integration.

In contrast, a standard MCMC approach requires constructing M independent Markov chains with additional data augmentation techniques (Albert & Chib, 1993; Polson et al., 2013), each targeting $f(\boldsymbol{\theta}|\boldsymbol{Y}_{1:(t-1)}, y_{j_t}, \boldsymbol{\xi}^{(m)})$, with sampling only possible after all chains have reached convergence. This introduces a significant computational burden, making MCMC impractical for real-time item selection.

We evaluate the effectiveness of our direct sampling approach in enabling fully Bayesian online item selection. Unlike traditional methods that treat item parameters as fixed, the fully Bayesian approach explicitly accounts for their uncertainties, which can be substantial when the item response dataset is small or poorly calibrated. To illustrate, we generate a binary item response dataset $\boldsymbol{Y}$ with $N = 500$ examinees and $J = 150$ items under a 3-factor probit MIRT model. The true factor loading matrix $\boldsymbol{B}$ has dimensions $J \times 3$, and the intercept vector $\boldsymbol{D}$ has 150 elements.

To integrate item parameter uncertainty into online item selection, we fit a Gibbs sampler to the $N \times J$ item response data. We generated $5,000$ MCMC draws, retaining the last 500 posterior samples of the item parameters $\Xi = \{\boldsymbol{\xi}^{(m)}\}_{m=1}^{500}$ after burn-in.

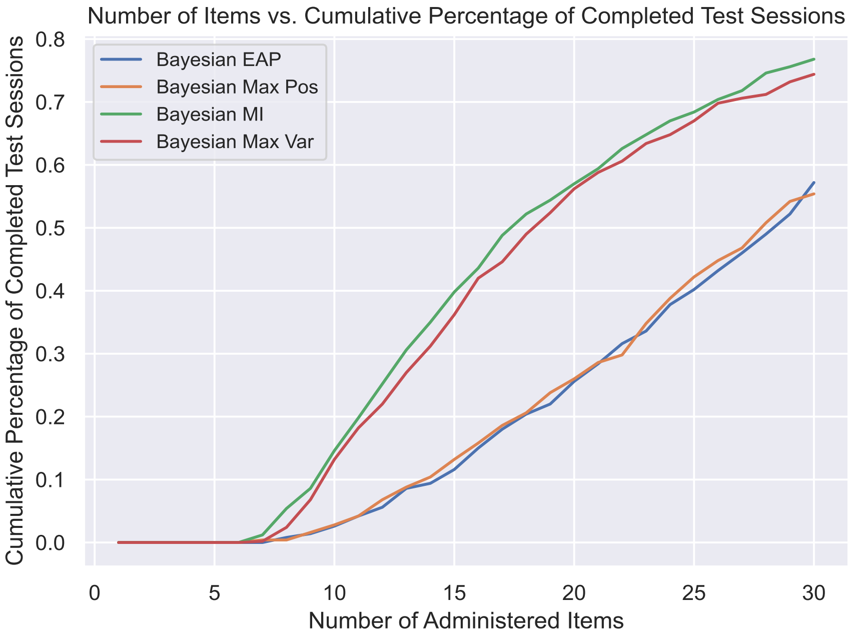

Figure 6: Fully Bayesian: Number of Items Versus Posterior Variance Reduction

As described in Section C.1, the fully Bayesian approach marginalizes over the joint distributions of the nuisance item parameters Ξ and latent traits. We then implemented all item selection rules depicted in Sections 3.2 in a fully Bayesian manner.

Figure 6 illustrates the cumulative percentage of completed test sessions across all 500 simulated examinees as more items are administered. The test was dynamically terminated when the posterior standard deviations across all three factors fell below 0.4. Consistent with the experimental results in Section 6 of the manuscript, the mutual information method and our proposed Max Var method demonstrated superior posterior variance reduction. The second column of Table 5 shows that the mutual information method terminated after an average of 18.7 items, whereas the EAP approach required 24.4 items.

Beyond variance reduction, the mutual information and maximizing prediction variance methods also exhibited superior estimation accuracy, as shown in Figure 7, which

Table 5: **Comparison of Winshares (W.S), Termination, and Computation**

**Algorithm**	**Avg Termination (items)**	**W.S dim0**	**W.S dim1**	**W.S dim2**	**Avg Time (s/item)**
EAP	24.4	22.2%	19.2%	21.8%	4.10
Max Pos	24.2	17.4%	18.0%	21.0%	4.10
MI	**18.7**	**32.8%**	31.0%	27.8%	4.11
Max Var	19.2	27.6%	**31.8%**	**29.4%**	4.10

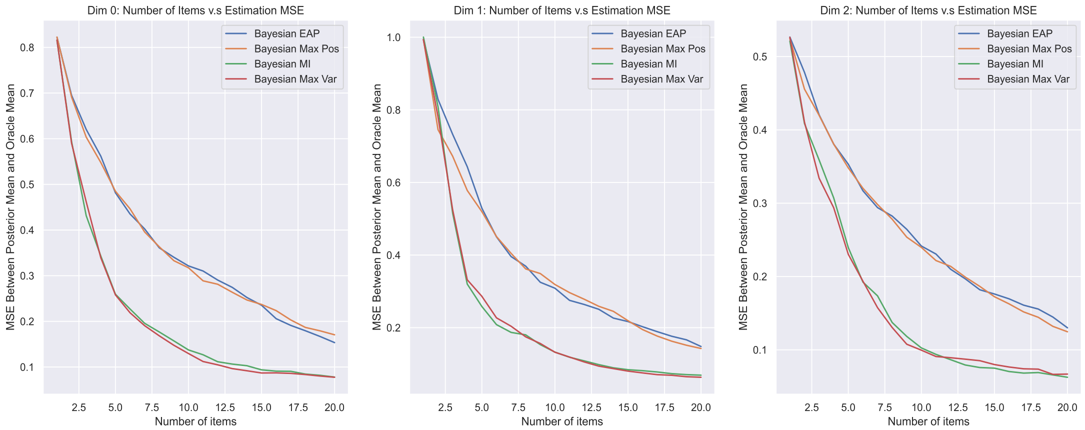

Figure 7: Fully Bayesian: MSE Between Posterior Mean and Oracle Posterior Mean

tracks the decline in mean squared error (MSE) between the posterior mean and the oracle posterior mean. Since estimation errors stabilized around $H = 30$ items, we compared MSEs between the posterior mean at $H = 20$ and the oracle posterior mean, and then summarized the win shares for each item selection rule across all 500 examinees and all three dimensions in Table 5.

Finally, we emphasize that even in a fully Bayesian framework, where nuisance parameters are integrated rather than fixed, our approach remains computationally efficient. Across all item selection rules, online selection required an average of just 4.1 seconds per test session on a personal laptop, highlighting the scalability of our direct sampling method for Bayesian MCAT applications. Readers may notice that 4.1 seconds is significantly longer than the item selection time reported in the experimentation section, where item parameters are treated as fixed. This discrepancy arises because

fully Bayesian MCAT requires sampling from $M = 500$ distinct unified-skew normal distributions for each item selection, whereas the standard case involves sampling from a single fixed distribution. This also explains the minimal differences in item selection time across algorithms, as the primary computational bottleneck lies in sampling from these distributions. When more CPUs are available, we can certainly sample from all M distributions in parallel, and hence further improving the 4.1 seconds benchmark.

D. Neural Network Design Details

Both the primary and target Q–networks share the same modular feed-forward design. At each time t, the posterior parameters $\tilde{\xi}_t$ are split into two streams: a permutation-invariant path ϕ_1 (three 256-unit ReLU layers) that processes the main state input. In parallel, a RowwiseNetwork $\phi_2()$ network applies independently to each of the J prediction-quartile feature vectors yields item-wise predictive values, which are then mapped to 256 dimensional features. The two 256-dimensional vectors are then merged by a small combiner MLP, which is then mapped to the final layers with J nodes through the classification network ρ. All linear layers use Xavier initialization; activations are ReLU; the final output is masked to $-\infty$ for unavailable items.

E. Discussion on the 0-1 Reward

Recall that our choice of reward function is defined in Equation (3.7). The simple 0-1 reward structure offers several advantages. It is interpretable as it directly minimizes the number of items required to terminate assessment tasks and prioritizes the main factors of interest. Additionally, it has demonstrated success in related applications, such as in designing deep adaptive learning system (X. Li et al., 2023). Since the cumulative reward is constrained to integer values between $-H$ and -1, the action-value function can be more reliably approximated by the neural network.

Table 6: Layer-by-layer specification of `OnlineQNetworkV` (and its submodules).

Module	Input Dim	Output Dim	Activation
ϕ_1 (Phi1Network)	S	256	ReLU
	256	256	ReLU
	256	256	ReLU
ϕ_2 (RowwiseNetwork) (for $\mathbf{\Psi}_t$)	11	256	ReLU
	256	256	ReLU
	256	1	Linear $\times J$ rows
ϕ_2 (SmallPhi2Network)	J	256	ReLU
	256	256	ReLU
	256	256	Linear
ρ (Classification)	256	J	ReLU
	J	J	Linear

Notes:

- S = total scalar-state dimension; Each new item selection introduced $(K + 2)$ dimensional tuple
- The RowwiseNetwork applies its three layers *independently* to each of the J rows of the $(J \times 11)$ $\mathbf{\Psi}_t$ matrix. There are 11 columns, because we include mean, variance, and the 10% quantile increments from 10% to 90%. Because we process each row independently, the output for the RowwiseNetwork is J dimensional.
- All weight matrices are initialized with Xavier uniform; no dropout is used.
- Optimizer: Adam with learning rate 10^{-4}, $\beta_1 = 0.9$, $\beta_2 = 0.999$.

Our double Q-learning algorithm enjoys theoretical convergence guarantees to the optimal policy (van Hasselt et al., 2016) and can accommodate arbitrary reward specifications. It is known that myopic policy cannot outperform the principled optimal policy obtained through RL. However, under certain reward structures, a greedy one-step lookahead (myopic) policy can perform surprisingly well.

In Chan and Farias (2009), it is shown that for stochastic depletion problems, a myopic policy achieves at least 50% of the expected reward of the optimal policy. A similar result can be proven for CAT under specific reward structures. Although this can be interpreted as a somewhat negative result for the reinforcement learning approach, we emphasize our choice of the 0-1 reward does not conform to the stochastic depletion problem, and hence is not applicable to the theorem below.

To illustrate this, we extend the state space $\mathcal{S}$ with a time variable, defining $\overline{\mathcal{S}} = \{(f(\boldsymbol{\theta} \mid \boldsymbol{Y}_{1:T}), t) : t \in [H]\}$. Let $\pi^*(\overline{s})$ represent the optimal policy, and $\pi^m(\overline{s})$ the myopic policy that maximizes the next-step expected reward. Define the mapping $\hat{G}_{y_a} : \overline{s} \longrightarrow \overline{s'}$ as the posterior update after observing the binary response random variable y_a, with an incremented time step, where $\overline{s} = (f(\boldsymbol{\theta}|\boldsymbol{Y}_{1:T}), t)$ and $\overline{s'} = (f(\boldsymbol{\theta}|\boldsymbol{Y}_{1:T}, y_a), t+1)$. Similarly, define $\tilde{G}_{y_a} : \overline{s} \longrightarrow \overline{s'}$ as the posterior update after observing y_a while keeping the time step fixed, where $\overline{s} = (f(\boldsymbol{\theta}|\boldsymbol{Y}_{1:T}), t)$ and $\overline{s'} = (f(\boldsymbol{\theta}|\boldsymbol{Y}_{1:T}, y_a), t)$.

To demonstrate the near-optimality of the myopic policy, Chan and Farias (2009) introduces two key assumptions: “value function monotonicity" and “immediate rewards". These assumptions can be adapted to the CAT setting as follows:

- **Immediate Rewards:** for any time stamp $t < H$, suppose the myopic policy π^m chooses item a, then we must have

$$V_{\pi^*}(\overline{s}) \leq E[R(\overline{s}, a, \overline{s}')] + V_{\pi^*}(\tilde{G}_{y_a}(\overline{s})). \tag{E.1}$$

  Essentially, the property says given a free item (without increasing the time step) chosen by the myopic policy would not deteriorate the optimal rewards.

- **Value Function Monotonicity:** for any time stamp $t < H$, suppose the optimal policy would choose item a_1 yet the myopic policy would chooses item a_2, then

$$V_{\pi^*}(\hat{G}_{y_{a_2}}(\tilde{G}_{y_{a_1}}(\overline{s}))) \leq V_{\pi^*}(\hat{G}_{y_{a_2}}(\overline{s})). \tag{E.2}$$

  Note both sides of inequality refer to the optimal value function evaluated at the posterior distribution at time step $t + 1$.

In Theorem E.1, we establish that a reinforcement learning approach to CAT cannot significantly outperform the one-step-lookahead greedy policy when the reward function satisfies the two assumptions outlined above.

Theorem E.1. *Consider the finite horizon CAT problem with H steps. Suppose the assumptions of immediate rewards in (E.1) and value function monotonicity in (E.2) hold, we have* $V_{\pi^*}(\overline{s}) \leq 2V_{\pi^m}(\overline{s})$ *for all* $\overline{s} \in \overline{\mathcal{S}}$.

Proof. We proceed by induction. Since the myopic and the optimal policy would agree on time $t = H - 1$, the inequality holds trivially for the base case. For the induction step, we assume the claimed inequality holds for all t' such that $H > t' > t$. Consider the time horizon $t < t'$ and let a_1 represent the item chosen by the optimal policy and a_2 the item chosen by the myopic policy. The inequality holds trivially for $a_1 = a_2$. Hence, we consider the case when $a_1 \neq a_2$ and let y_{a_1} and y_{a_2} represent the binary random variables for the item response. For given extended state $\overline{s}$, we use $\overline{s'}$ to denote the next step after selecting an item. Then we have

$$
\begin{aligned}
V_{\pi^*}(\overline{s}|y_{a_1}, y_{a_2}) &= E[R(\overline{s}, a_1, \overline{s'})|y_{a_1}] + V_{\pi^*}(\hat{G}_{y_{a_1}}(\overline{s})) \\
&\leq E[R(\overline{s}, a_1, \overline{s'})|y_{a_1}] + E[R(\overline{s}, a_2, \overline{s'})|y_{a_2}] + V_{\pi^*}(\tilde{G}_{y_{a_2}}(\hat{G}_{y_{a_1}}(\overline{s}))) \qquad \text{(E.3)} \\
&= E[R(\overline{s}, a_1, \overline{s'})|y_{a_1}] + E[R(\overline{s}, a_2, \overline{s'})|y_{a_2}] + V_{\pi^*}(\hat{G}_{y_{a_1}}(\tilde{G}_{y_{a_2}}(\overline{s}))) \\
&\leq E[R(\overline{s}, a_1, \overline{s'})|y_{a_1}] + E[R(\overline{s}, a_2, \overline{s'})|y_{a_2}] + V_{\pi^*}(\hat{G}_{y_{a_2}}(\overline{s}))) \qquad \text{(E.4)} \\
&\leq E[R(\overline{s}, a_1, \overline{s'})|y_{a_1}] + E[R(\overline{s}, a_2, \overline{s'})|y_{a_2}] + 2V_{\pi^m}(\hat{G}_{y_{a_2}}(\overline{s}))). \qquad \text{(E.5)}
\end{aligned}
$$

Note we applied the immediate rewards assumption in equation (E.3), the value function monotonicity assumption in equation (E.4), and the induction hypothesis in equation (E.5). Now, we may take expectation over y_{a_1} and y_{a_2} on both side of inequality and

obtain:

$$V_{\pi^*}(\overline{s}) \le E[R(\overline{s}, a_1, \overline{s'})] + E[R(\overline{s}, a_2, \overline{s'})] + 2V_{\pi^m}(\hat{G}_{y_{a_2}}(\overline{s}))$$
$$\le 2(E[R(\overline{s}, a_2, \overline{s'})] + V_{\pi^m}(\hat{G}_{y_{a_2}}(\overline{s}))) = 2V_{\pi^m}(\overline{s}).$$

The last inequality is due to the fact the π^m is the myopic policy that maximizes the next step reward. □

Theorem E.1 can be seen as a limitation of the reinforcement learning approach when the reward structure aligns with the stochastic depletion problem, as it implies that a simple myopic policy provides a 2-approximation to the optimal policy. However, it also highlights the advantage of the simple 0-1 reward function as the value function monotonicity is unlikely to hold, rendering the theorem inapplicable. Specifically, the expected posterior variance of $\hat{G}_{y_{a_2}}(\tilde{G}_{y_{a_1}}(\overline{s}))$ is expected to be smaller than that of $\hat{G}_{y_{a_2}}(\overline{s})$. Consequently, fewer items are expected for the posterior $\hat{G}_{y_{a_2}}(\tilde{G}_{y_{a_1}}(\overline{s}))$ to reach the minimum variance threshold, which implies $V_{\pi^*}(\hat{G}_{y_{a_2}}(\tilde{G}_{y_{a_1}}(\overline{s}))) \ge V_{\pi^*}(\hat{G}_{y_{a_2}}(\overline{s}))$.

F. More Analysis on Simulation

F.1. Factor Loading Matrix Visualization

We visualize the factor loading matrix in our simulation exercise in Figure 8. Note all the factor loadings are bounded between $(-3, 3)$ and the matrix is relatively sparse: all the items are loaded on the first factor, but are only allowed to load for at most two more factors. Although identification of the factor loading matrix is not a primary concern in CAT, as it is typically treated as fixed, we purposefully imposed a lower-triangular structure to ensure identifiability and create a more realistic simulation setting.

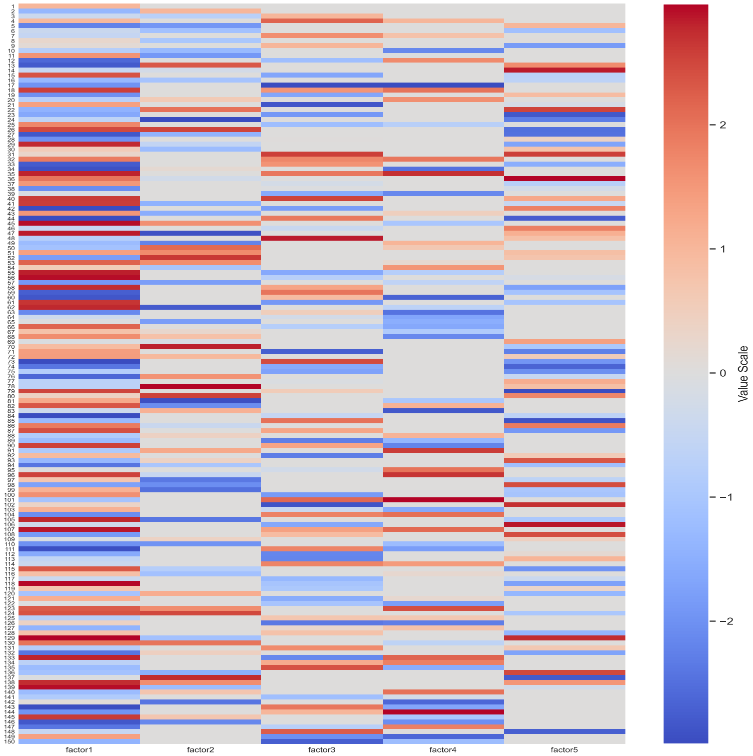

Figure 8: Simulated Factor Loading Matrix

F.2. Double Q-Learning Training Dynamics

In Figure 9, we present a metric illustrating the training dynamics of our Q-learning algorithm. The left subplot displays the average episode rewards, computed over every 500 training episodes, showing that rewards began converging around episode 25,000 at approximately -23. The reward is bounded within $[-40, -20]$. Given our definition of the $0-1$ reward, the implication is that a random policy would take 40 items for the test to converge. After training, it would only take around 20 items.

Since we save the primary neural network every 1000 episodes, we select the checkpoint corresponding to the highest average reward for offline deployment in future item selection tasks. In this case, the network at episode 72,000 is chosen, as it

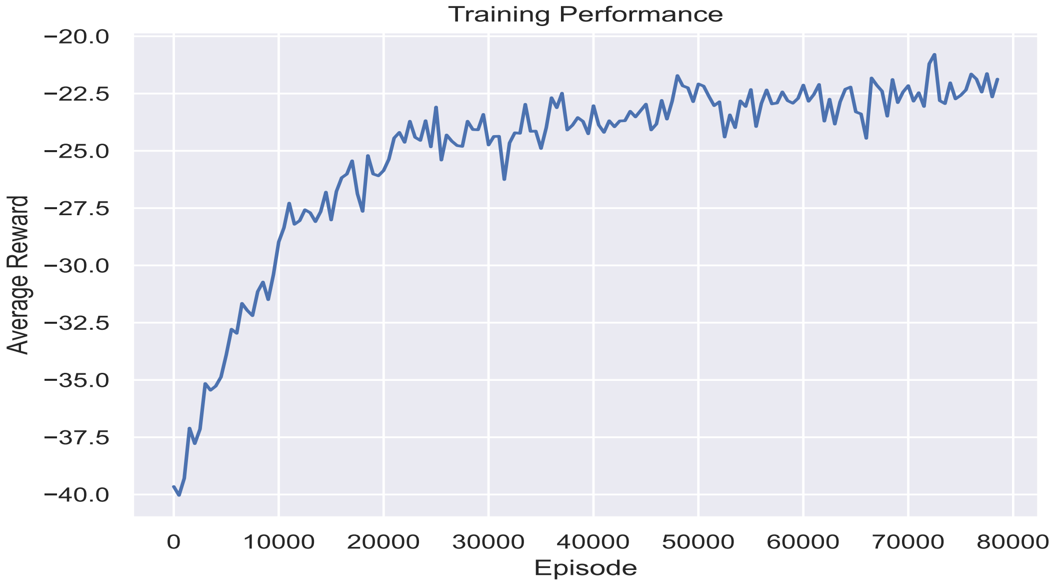

Figure 9: Training Dynamics for the Simulation Experiment in Section 5.1

achieves the peak average reward of approximately -21. This model selection strategy is consistently applied across all experiments in Section 5.

F.3. Matching the Oracle Posterior

Recall the "oracle posterior distribution" of a test taker is defined as the posterior obtained if a given test taker had answered all items in the item bank. To obtain the oracle distributions for each of the 500 test takers in Section 6, we simulate their item response to all 150 items in the test bank, and then calculate the posterior distribution using Theorem 4.1.

Figures 10, 12, and 13 illustrate the decrease of the estimation MSE for the first quartile, median, and the third quartiles compared to those of the oracle distribution, respectively. The quartiles of each posterior distribution are computed via $1,000$ independent draws. In all these figures, we observe similar superior performances for the MI, Max Var, and the Q-learning methods over the EAP and the Max Pos approaches

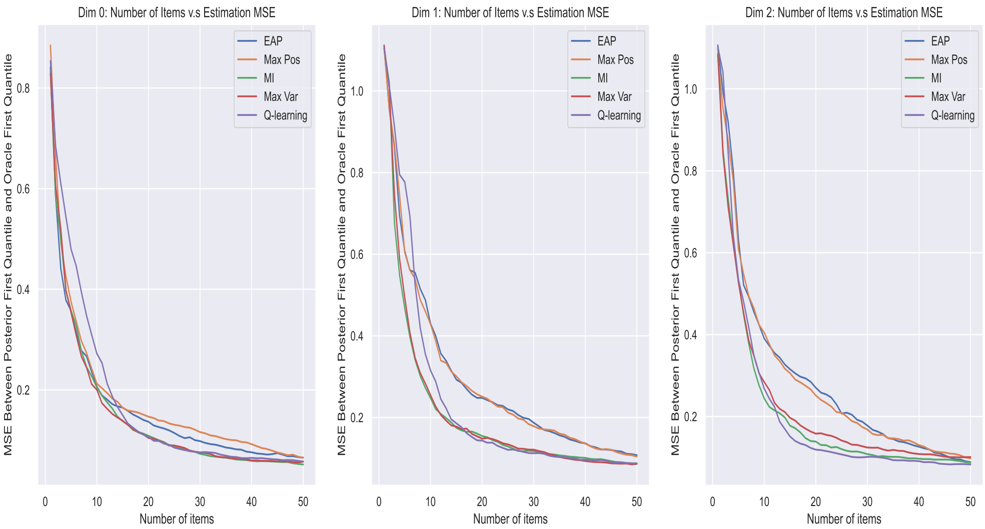

Figure 10: MSE Between Posterior 1st Quantile and Oracle 1st Quantile

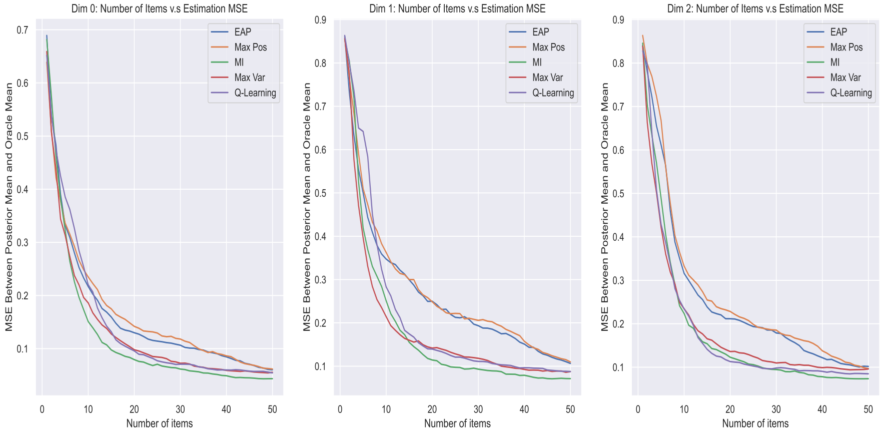

Figure 11: MSE Between Posterior Means and Oracle Posterior Means

in estimating the entire oracle distributions. Consistent with the findings in Section 6, the Q-learning approach demonstrates the fastest error reduction rate, especially in the early stages of the test.

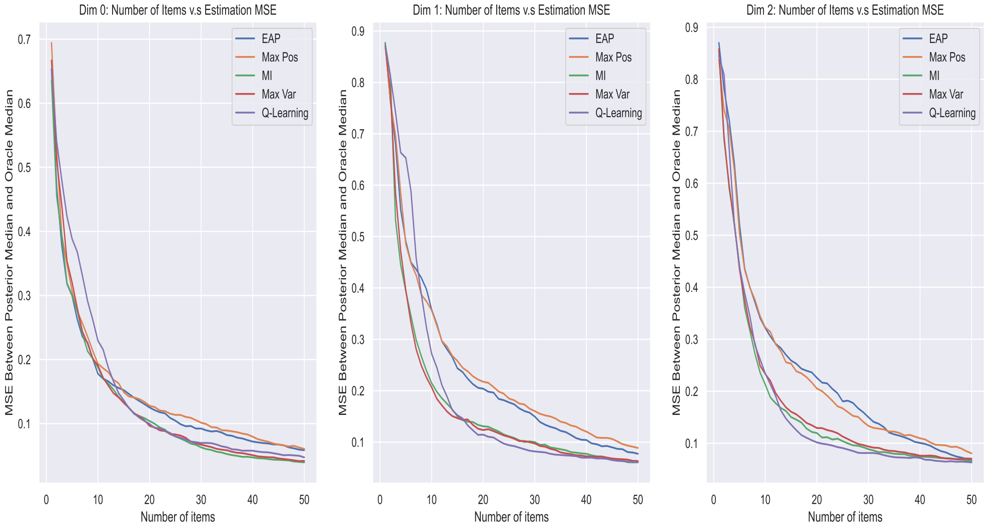

Figure 12: MSE Between Posterior Median and Oracle Median

G. pCAT-COG Study Additional Visualization

G.1. Exposure rate

In the real-data experiment, we administer the full 57-item bank in every simulated session to compare the rate of MSE reduction as items accrue. Consequently, item exposure is trivially 100% for all items under all methods, and exposure-rate comparisons are not directly informative in this setting. Therefore, we visualize the distributions of item exposure rate when $T = 30$ in Figure 14. Since the full item bank has 57 items, we should expect the exposure rate to be centered around $30/57 \approx 52.6\%$.

G.2. Q-learning Training Dynamics

H. DESE Educational Assessment

We consider an important application in educational assessment, utilizing 2022 Grade 8 student item response data from the Massachusetts Department of Elementary and Secondary Education (DESE). Excluding non-multiple-choice questions, the dataset

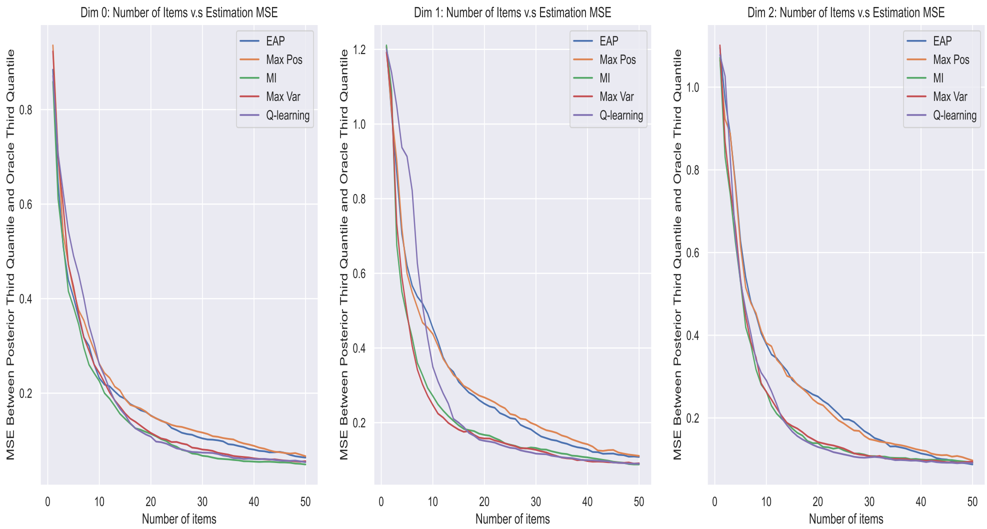

Figure 13: MSE Between Posterior 3rd Quantile and Oracle 3rd Quantile

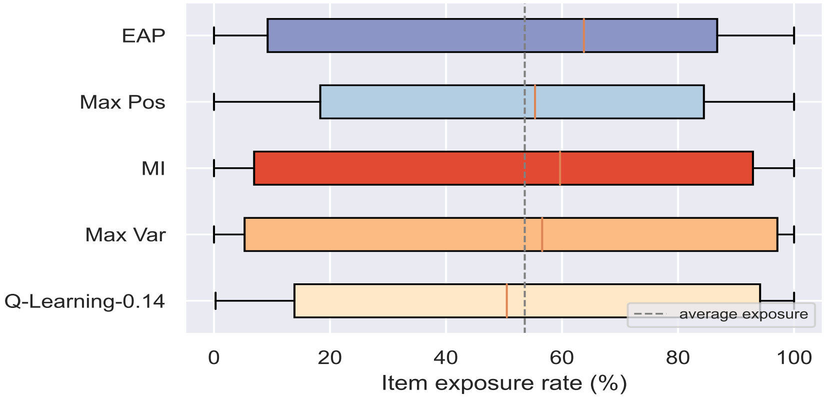

Figure 14: Distributions of Item Exposure Rates at $T = 30$: pCAT-COG

includes responses to 24 English items and 34 math items. For efficiency, we randomly sampled $1,000$ student responses to evaluate each CAT item selection algorithm.

Given the unknown latent structure, we estimate the factor loading matrix via the PXL-EM algorithm (J. Li et al., 2025), resulting in a 58 by 4 factor loading matrix, with visualization and the details of model fitting provided in Appendix H. When estimating the item parameters, we adopted the idea of dynamic posterior exploration, letting the

regularization λ_0 parameter in the PXL-EM algorithm to gradually increase over the path $\{1, 5, 10, 20, 40, 60, 80, 100\}$. The resulting factor loading matrix $\boldsymbol{B}$ is visualized in Figure 16, where dark black regions indicate exact zeros. Notably, all English items load exclusively onto the first factor, representing general ability, while math items can also load onto factors 2–4, reflecting their greater structural complexity. For instance, the final factor ("Math 3") has nonzero loadings only on items 34, 36, and 38, all of which correspond to geometry problems. [1] Interestingly, the authors in J. Li et al., 2025 obtained very similar factor loading matrix estimation for the Grade 10 data as well.

Since all items are heavily loaded on the first primary factor, representing the global latent ability, we focus on the problem of adaptively measuring the first factor while accounting for the underlying multidimensional structure in dimensions 2-4. The left subplot of Figure 15 illustrates the cumulative percentage of completed test sessions for the $1,000$ sampled examinees as more items are administered. As shown in the plot, nearly 70% of CAT sessions terminate after only 20 items under the Q-learning policy. On average over these $1,000$ examinees, Q-learning also required only 20 items to reach the termination criterion, whereas the second-fastest method (MI) required nearly 26 items. Beyond its efficiency in variance reduction, Q-learning also achieves a faster decay in MSE when estimating the first factor, reinforcing findings from earlier experiments.

These results highlight the potential of Q-learning for educational assessments, demonstrating its ability to adapt to various testing environments and to accelerate testing while maintaining accurate estimation of students' multivariate latent traits.

[1] Exam questions are available at: https://www.doe.mass.edu/mcas/2022/release/.

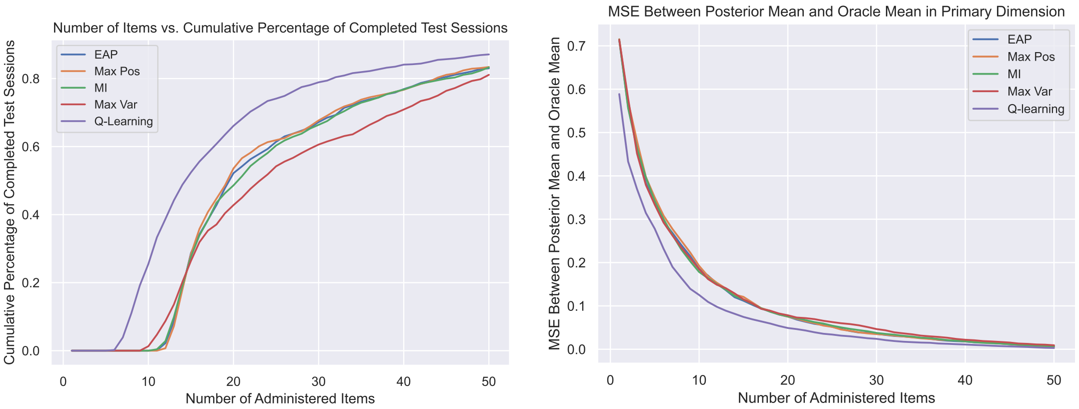

(a) Test Termination Speed

(b) Test MSE Accuracy

Figure 15: Educational Assessment: Main Factor Posterior Variance Reduction (Left) and Estimation Accuracy (Right)

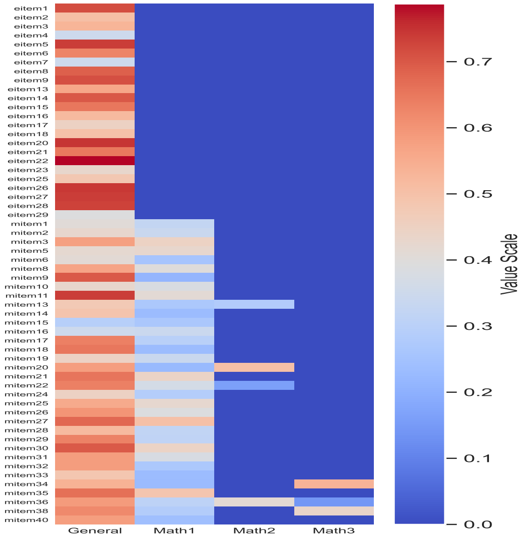

Figure 16: Estimated Factor Loadings for DESE Data
